# Carboxypeptidase D deficiency causes hearing loss amenable to treatment

**DOI:** 10.1172/JCI192090

**Published:** 2025-09-30

**Authors:** Memoona Ramzan, Natalie Ortiz-Vega, Mohammad Faraz Zafeer, Amanda G. Lobato, Tahir Atik, Clemer Abad, Nirmal Vadgama, Duygu Duman, Nazım Bozan, Enise Avcı Durmuşalioǧlu, Sunny Greene, Shengru Guo, Suna Tokgöz-Yılmaz, Merve Koç Yekedüz, Fatma Tuba Eminoğlu, Mehmet Aydın, Serhat Seyhan, Ioannis Karakikes, Vladimir Camarena, Maria Camila Robayo, Tijana Canic, Güney Bademci, Gaofeng Wang, Amjad Farooq, Mei-ling Joiner, Katherina Walz, Daniel F. Eberl, Jamal Nasir, R. Grace Zhai, Mustafa Tekin

**Affiliations:** 1 John P. Hussman Institute for Human Genomics and; 2 Department of Molecular and Cellular Pharmacology, University of Miami Miller School of Medicine, Miami, Florida, USA.; 3 Department of Neurology, University of Chicago, Chicago, Illinois, USA.; 4 Division of Pediatric Genetics, Department of Pediatrics, School of Medicine, Ege University, Izmir, Turkey.; 5 Department of Cardiothoracic Surgery, Stanford University, Stanford, California, USA.; 6 Department of Audiology, Ankara University Faculty of Health Sciences, Ankara, Turkey.; 7 Ankara University Rare Diseases Application and Research Center, Ankara, Turkey.; 8 Department of Otolaryngology, Faculty of Medicine, Yüzüncü Yıl University, Van, Turkey.; 9 Division of Pediatric Metabolic Diseases, Department of Pediatrics, Ankara University School of Medicine, Ankara, Turkey.; 10 Harvard Medical School, Boston Children’s Hospital, Department of Anesthesiology, Critical Care and Pain Medicine, Boston, Massachusetts, USA.; 11 Laboratory of Genetics, Memorial Şişli Hospital, Istanbul, Turkey.; 12 Dr. John T. Macdonald Foundation Department of Human Genetics and; 13 Department of Biochemistry, University of Miami Miller School of Medicine, Miami, Florida, USA.; 14 Department of Biology, University of Iowa, Iowa City, Iowa, USA.; 15 Instituto de Química Biológica de la Facultad de Ciencias Exactas y Naturales (IQUIBICEN) CONICET, Buenos Aires, Argentina.; 16 Division of Life Sciences, University of Northampton, Northampton, United Kingdom.

**Keywords:** Genetics, Otology, Genetic diseases

## Abstract

Genetic factors contributing to hearing loss (HL) are heterogeneous, and effective medical treatments remain limited. We identified 3 distinct missense variants in *CPD*, encoding carboxypeptidase D, in 5 individuals with congenital deafness from 3 unrelated families, affecting the catalytically active CP domain 2 of this protein. Subsequent analysis of a larger cohort from the 100,000 Genomes Project revealed an enrichment of rare protein-altering *CPD* variants in individuals with HL. We show that CPD localizes to sensory epithelium and nerve cells in the mouse cochlea, and the enzymatic activity of CPD, crucial for nitric oxide (NO) production through arginine processing, is impaired in affected individuals. The levels of arginine, NO, and cGMP in patient-derived fibroblasts are also decreased, leading to endoplasmic reticulum stress–mediated responses being triggered in the cells. Silencing of *Cpd* in organotypic mouse cochlea cultures leads to increased apoptosis. Finally, *Drosophila* models of CPD deficiency display defective Johnston’s organ, impaired auditory transduction, and sensory and movement abnormalities. Notably, these phenotypes are partially rescued by supplementation with arginine or sildenafil, a cGMP enhancer. Our findings establish *CPD* mutations as a cause of congenital HL, highlighting that the NO signaling pathway offers a promising therapeutic avenue.

## Abstract 2

Changes in the CPD gene cause hearing loss by harming inner ear cells. Restoring a natural chemical signal with nutrients like arginine may improve hearing.

## Graphical abstract

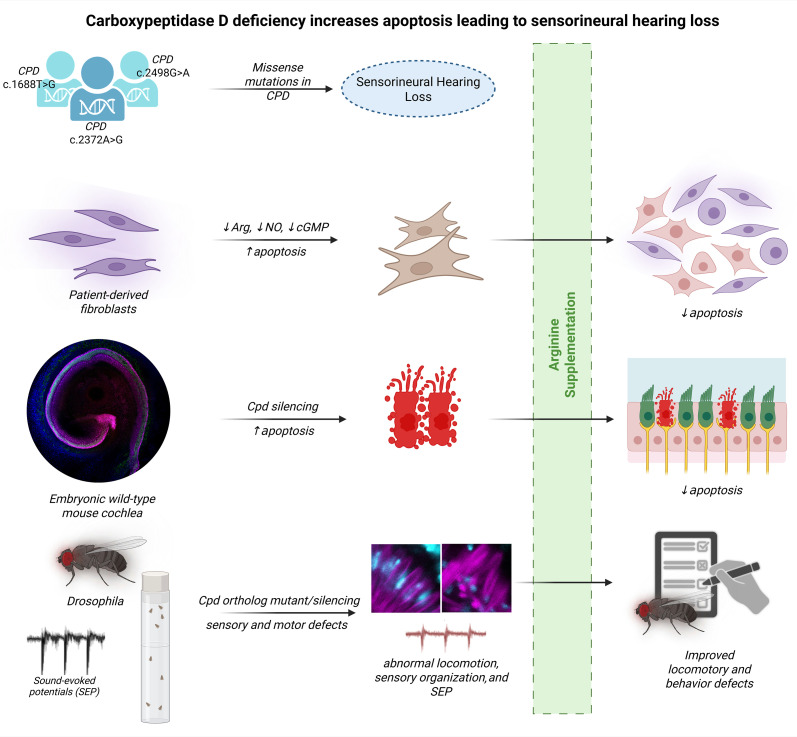

## Introduction

Hearing loss (HL) affects approximately 1 in 500 newborns and becomes increasingly common with age, affecting nearly half of adults over 65 years old (1, 2). It often results from dysfunction in the delicate structures or complex signaling pathways of the cochlea (3). Among the recognized auditory pathways, the role of nitric oxide (NO) signaling is not thoroughly characterized. Conventionally, NO is synthesized from arginine and oxygen in the presence of NO synthase (NOS) and cofactors (4). The arginine used as a substrate to produce NO can be generated by metallocarboxypeptidases, a group of zinc-dependent enzymes involved in the catalytic cleavage of proteins at the carboxyl terminus (5). Carboxypeptidase D (CPD) is an important member of this enzyme family and possesses the ability to cleave peptides with the C-terminal arginine or lysine (5).

Previous studies have shown that NO signaling is involved in sensory hair cells, supporting cells, and spiral ganglion in the cochlea (6–10), as well as in the brainstem, where the cochlear nucleus is located (11–15). Perturbation of NO signaling has been implicated in endoplasmic reticulum (ER) stress and mitochondrial dysfunction across various diseases (16–18). However, the integrated role of NO in hearing remains poorly understood, and no hereditary form of deafness has been directly linked to mutations in components of the NO signaling pathway.

Sensorineural hearing loss (SNHL) is the most common type of permanent HL, caused by damage to the cochlea or the auditory nerve (19). SNHL is generally irreversible, and although hearing aids and cochlear implants are commonly used to improve hearing, they do not completely restore physiological functioning. Recently, the first gene therapy clinical trials for hereditary deafness have shown promising results (20). However, medical treatment for hereditary deafness is not readily available.

Our study identifies CPD as a critical regulator of metabolic signaling, linking arginine and NO to cochlear cell survival. We describe 3 unrelated families with *CPD* variants identified via genome sequencing (GS) and exome sequencing (ES). Our in vitro and in vivo functional analyses show that dysfunction of *CPD* causes NO deficiency and apoptosis, which can potentially be treated using dietary supplements rich in arginine.

## Results

### CPD variants in 3 families with deafness.

**Figure 1:**
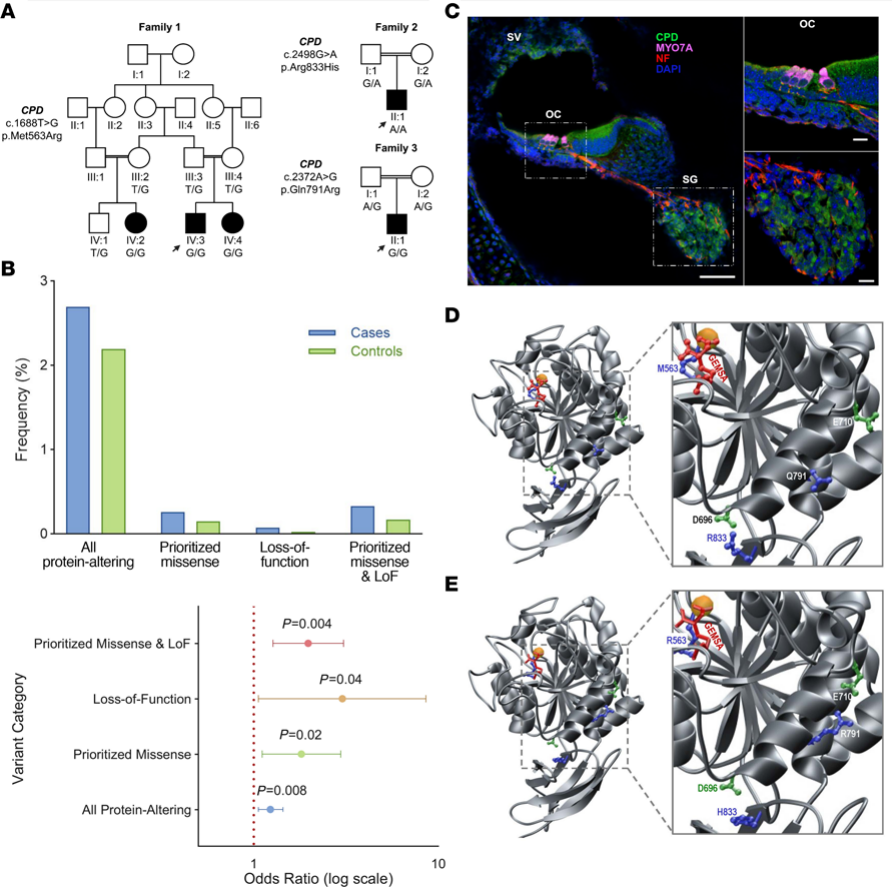
Pedigrees, *CPD* variant enrichment in HL population, protein localization, and in silico modeling. (**A**) Pedigrees of 3 participating families with variant segregation are shown. Males are represented by squares and females by circles; filled symbols indicate affected individuals; and double lines denote consanguinity. Arrows mark probands. (**B**) Comparison of the frequency of disease-causing variants in CPD between hearing loss (HL) cohorts and controls reveals a higher occurrence of protein-altering (2.69% vs. 2.19%), missense (0.26% vs. 0.15%), and loss-of-function (LoF) variants (0.07% vs. 0.02%) in patients, suggesting a stronger contribution of these variants to HL. The forest plot displays ORs with 95% CIs for disease-causing CPD variants. The red dotted line (OR = 1) represents no association. ORs were significantly elevated for combined variants (OR = 1.97), LoF variants (OR = 3.02), and prioritized missense variants (OR = 1.81), confirming a strong association between CPD variants and HL. (**C**) CPD localization in the mouse inner ear. Representative staining of P0 cochlear sections shows CPD (green), MYO7A (magenta), neurofilament (red), and DAPI (blue). The right panels show magnified views of the boxed regions. Spiral ganglion (SG), organ of Corti (OC), and stria vascularis (SV) are labeled. CPD localizes prominently to the SG, SV, and OC. Scale bar: 60 μm; right panels: 15 μm. (**D** and **E**) Ribbon representation of the structural model of the wild-type protein and the variant harboring the M563R/Q791R/R833H triplet of human CP domain 2 (residues 501–875). CP domain 2 comprises 2 subdomains: an N-terminal αβ-fold harboring a Zn^2+^ divalent ion (yellow sphere) as a cofactor at the heart of the active site, followed by a C-terminal β-barrel. The protein backbone is gray; side chains of the M563R/Q791R/R833H triplet are blue, with interacting partners in green. The peptidomimetic inhibitor GEMSA is depicted in red.

The families reported here are a part of an international cohort comprising 1,012 families with SNHL. Among these, 441 families remained unsolved after exclusion of single-nucleotide variants, small insertions and deletions, and copy number variants in all known deafness genes via ES and GS. Affected individuals in 3 Turkish families presented in this study have congenital or prelingual-onset, bilateral severe-profound or profound SNHL (Figure 1A and Supplemental Figure 1; supplemental material available online with this article; https://doi.org/10.1172/JCI192090DS1). The detailed systemic physical examinations in the affected individuals showed no other clinical findings associated with HL, and gross vestibular function was normal (Table 1 and Supplemental Tables 1 and 2).

As detailed in Methods, we analyzed ES and GS data, focusing on rare variants cosegregating with HL in each family. The proband of family 1 is homozygous for a missense variant, c.1688T>G p.(Met563Arg), in *CPD* mapping to an autozygous region on chromosome 17 and cosegregating with HL within the extended family (Figure 1A, Table 1, Supplemental Figure 2A, and Supplemental Table 1). Further analysis via GS did not reveal any plausible coding/non-coding variants or copy number variants in recognized genes for deafness. Specifically, no pathogenic variants were identified in *MYO15A* or *GRAP*, which map to the same chromosomal region. In the probands of families 2 and 3, we identified the missense variants c.2498G>A p.(Arg833His) and c.2372A>G p.(Gln791Arg), respectively, in *CPD*. Both variants cosegregate with HL in an autosomal recessive pattern (Figure 1A, Table 1, Supplemental Figure 2A, and Supplemental Table 1).

### Functional CPD variants are enriched in HL probands as compared with controls.

To assess the broader impact of *CPD* variants at the population level and investigate whether they might contribute to HL later in life, we conducted a burden analysis using a large cohort from the 100,000 Genomes Project (100KGP) (21). It comprised 3,802 individuals with HL and 27,503 controls. This allowed us to determine the extent to which rare protein-altering variants influence disease susceptibility beyond the severe cases identified in family studies.

Owing to Genomics England policy, individual-level clinical data from 100KGP cannot be disclosed, and only aggregate summaries of phenotypic information are permitted. Nevertheless, most cases with rare *CPD* variants exhibited features highly consistent with the clinical profile observed in our familial cases, including congenital or childhood-onset bilateral SNHL, with severity ranging from severe to profound. Several individuals also underwent MRI of the internal auditory meatus, with findings consistent with primary inner ear dysfunction, in keeping with the expected cochlear and neuronal localization of *CPD*.

A subset of individuals displayed additional or potentially unrelated features, including middle ear pathologies such as otitis media or conductive HL. While these findings may suggest comorbid or acquired conditions rather than the primary consequence of *CPD* dysfunction, we retained these individuals in the broader analysis to avoid overly restrictive filtering that might obscure the contribution of *CPD* variants to a genetically heterogeneous clinical spectrum.

The analysis of all protein-altering variants in *CPD* showed an alternate allele frequency of 2.69% in cases and 2.19% in controls, yielding an odds ratio (OR) of 1.23 (95% CI: 1.06–1.44; *P* = 0.0084). This result indicates a statistically significant but modest enrichment of protein-altering variants in cases compared with controls (Figure 1B and Supporting Data Values Excel file).

For prioritized missense variants with a minor allele frequency less than 0.001 and either a Combined Annotation Dependent Depletion score ≥ 25 or an AlphaMissense score indicating probable damage or ambiguity (≥0.34), the alternate allele frequency was 0.26% in cases and 0.15% in controls, resulting in an OR of 1.81 (95% CI: 1.11–2.96; *P* = 0.0211). This suggests a statistically significant association, with prioritized missense variants being more frequent in cases. Furthermore, analysis of loss-of-function (LoF) variants revealed an alternate allele frequency of 0.07% in cases and 0.02% in controls. The OR was 3.02 (95% CI: 1.06–8.57; *P* = 0.0465), demonstrating a significant enrichment of LoF variants in cases (Figure 1B and Supporting Data Values Excel file). This category exhibited the strongest association among all variant groups.

The combined category of prioritized missense and LoF variants showed an alternate allele frequency of 0.33% in cases and 0.17% in controls. The OR was 1.97 (95% CI: 1.27–3.07; *P* = 0.0042), reflecting a statistically significant association, with these variants being more frequent in cases (Figure 1B).

### In silico modeling predicts reduced catalytic activity in CPD mutants.

CPD consists of three CP domains: extracellular N-terminal, cytosolic C-terminal, and a small transmembrane domain (Supplemental Figure 2B). All detected variants affect highly conserved amino acids localized in the catalytically active CP domain 2 (Supplemental Figure 2B).

CP domain 2 consists of an N-terminal αβ-fold harboring a Zn^2+^ divalent ion as a cofactor at the active site and a C-terminal β-barrel (Figure 1D). Met563 forms a part of a group of residues that give structural integrity to the catalytic center of the protein (Figure 1D). The p.(Met563Arg) variant would lead to distortion of the active site due to the bulky, basic side chain of arginine (Figure 1D). Moreover, it would also serve as a non-competitive inhibitor of potential substrates.

The side chain moiety of Gln791 is exposed to the solvent, but also, along with other polar residues, enables the protein to “breathe,” thereby enforcing rapid substrate turnover. The substitution of arginine for Gln791 leads to the formation of an additional salt bridge with the neighboring Glu710 (Figure 1E). The resulting molecular interaction causes structural disruption and forms an additional layer of rigidity to the protein, compromising its catalytic activity.

Arg833 forms a salt bridge with Asp696 (Figure 1E), which tethers the β-barrel to the back of the αβ-fold, away from its catalytic center, and is critical for overall structural integrity. The p.(Arg833His) variant would destabilize the protein and likely deactivate it by distorting its catalytic center (Figure 1D).

### CPD localization in mouse cochlea and antibody validation.

We detected *Cpd* mRNA expression in the cochlea of embryonic (E18.5), neonatal (P0), and adult mice (P15 and P30) via quantitative and semiquantitative reverse transcriptase PCR analysis. *Cpd* is consistently expressed across cochlear development and is present in multiple cochlear cell types (Supplemental Figure 3, A and B). Immunostaining with validated anti-CPD antibody (Supplemental Figures 4 and 5) on cochlear sections from wild-type mice shows localization of CPD in both inner and outer hair cells, stria vascularis, and spiral ganglion (Figure 1C and Supplemental Figure 4). This pattern is consistent mainly with public RNA-Seq and single-cell RNA-Seq (scRNA-Seq) datasets available via the gEAR portal (https://umgear.org), which show strong expression in the hair cells, stria vascularis, and spiral ganglion (Supplemental Figure 3, C and D). Together, these complementary data highlight the broad and sustained presence of CPD in the cochlea, supporting its relevance throughout development and into postnatal maturation.

### Reduced enzyme activity and altered protein length in CPD mutants.

**Figure 2:**
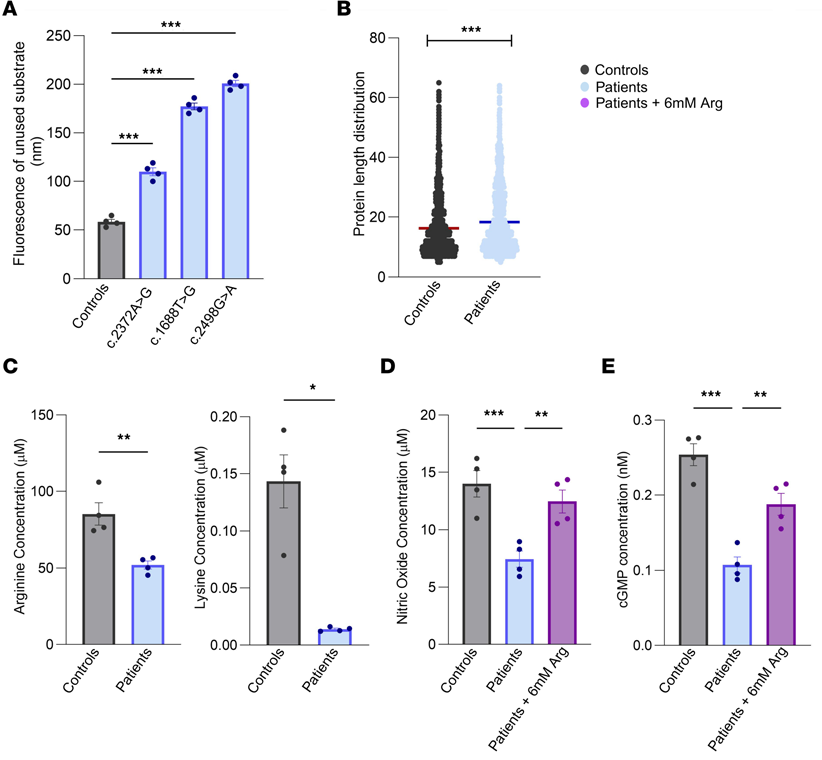
In vitro analysis of CPD variants. (**A**) Comparison of enzyme activity in mutated and control cells shows significantly reduced activity in patients, as evidenced by fluorescence-based substrate assays. (**B**) Mass spectrometry analysis revealed a difference in protein length between controls and patients in fibroblasts, with longer peptide lengths observed in patients. (**C**) Effect on the concentration of intracellular arginine and lysine in patient fibroblasts, compared with controls. (**D**) Nitric oxide (NO) concentration measured in controls, patients, and treated groups. (**E**) cGMP levels measured in controls, patients, and treated groups. The results are from 3–4 independent experiments, expressed as mean ± SEM. Statistical significance was determined using the unpaired 2-tailed *t* test and ANOVA. Significant differences are denoted with asterisks (*P ≤ 0.05, **P ≤ 0.001, ***P ≤ 0.0001) when compared with controls or between patients with treatment to the patients without treatment.

CPD is known to cleave the C-terminal Arg/Lys of its substrates. Thus, the disruption in the catalytic domain of the enzyme is expected to decrease the efficiency of peptide cleavage, resulting in longer peptides or unused substrate. It is evident in Figure 2A that when the lysate containing the mutated CPD was incubated with dansyl-Ala-Arg, a significant amount of the substrate was left uncleaved. This is supported by liquid chromatography–mass spectrometry data analysis, which reveals a relative increase in longer peptides from patients’ fibroblasts compared with the control group (Figure 2B).

### Low levels of lysine, arginine, NO, and cGMP in patient fibroblasts rectified by exogenous arginine.

The arginine cleaved by CPD could serve as a substrate for NO synthase to produce NO, which in turn signals various biological processes, including the conversion of GTP to cyclic guanosine monophosphate (cGMP) (22). To test this, we first measured the levels of intracellular arginine (and lysine) in control and patient fibroblasts. We observed a decrease in both amino acids in patient cells compared with control (Figure 2C and Supplemental Figure 6A). Further, NO and cGMP levels were also reduced in patient cells when compared with the control group (Figure 2, D and E, and Supplemental Figure 6, B and C). These results support the hypothesis that the disruption of the catalytic site of CPD leads to a reduction of intracellular arginine, lysine, and NO signaling.

We subsequently administered l-arginine to patient fibroblasts, which reversed the levels of intracellular NO and cGMP to almost normal levels, compared with the control groups (Figure 2, D and E, and Supplemental Figure 6, B and C). These results suggest that the identified variants cause damage to the catalytic domain of CPD, reduction of intracellular arginine, and defective NO signaling, which can be reversed with the supplementation of arginine.

### Decrease in NO levels causes apoptosis in fibroblasts.

**Figure 3:**
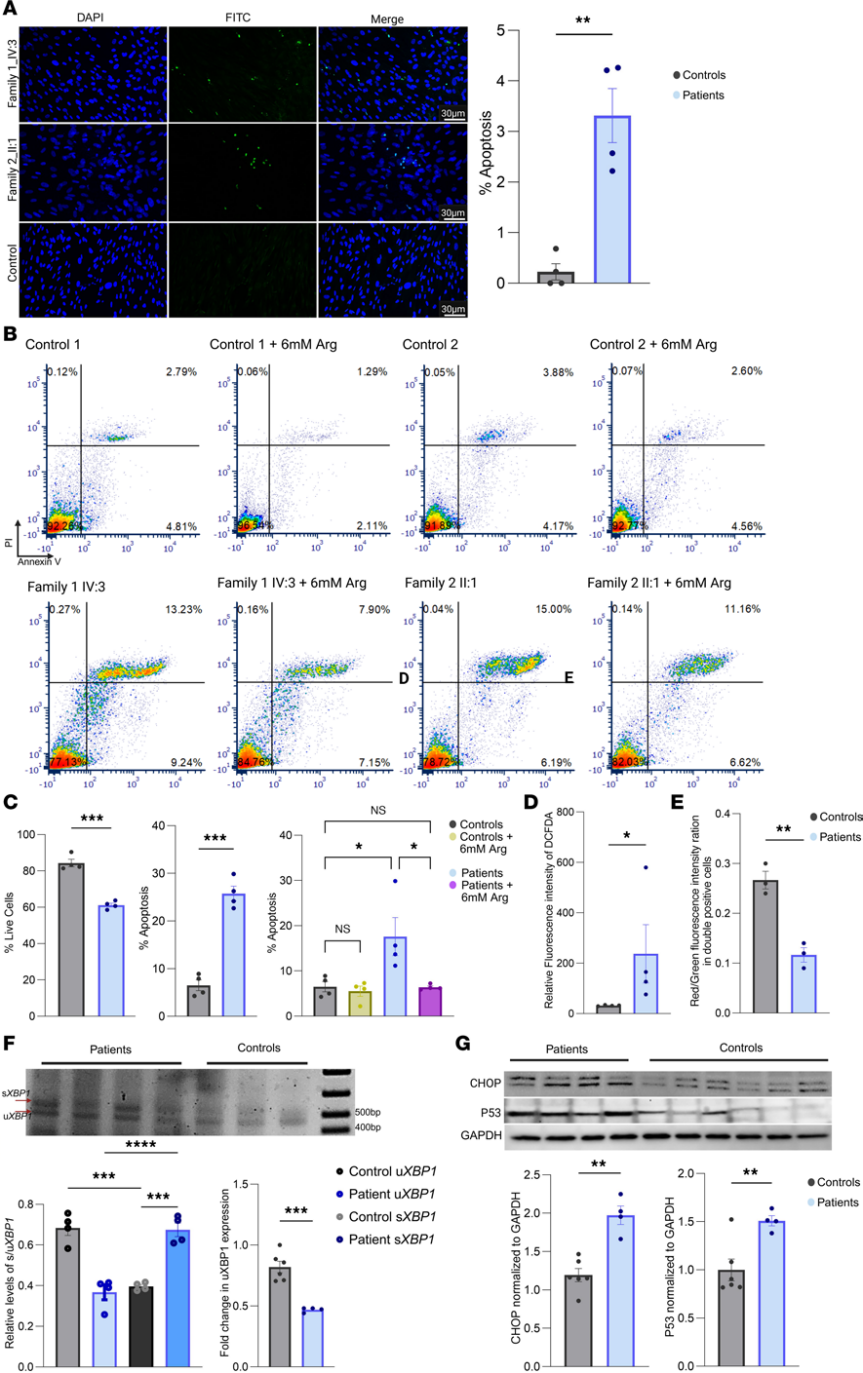
Analysis of apoptosis and mechanistic pathways in patient-derived fibroblasts. (**A**) Representative images of TUNEL staining showing increased apoptosis in patient-derived fibroblasts compared with controls, with quantification of apoptotic cells (% Apoptosis, right). Scale bars: 30 μm. (**B** and **C**) Representative images of flow cytometry (**B**) and statistical analysis (**C**) of annexin V and PI staining in patient fibroblasts (bottom row) compared with controls (top row). Arginine supplementation reduces apoptosis in patient fibroblasts, as shown in the quantification of live and apoptotic cells (far right in **C**). (**D**) Relative fluorescence intensity of H_2_DCFDA indicates higher levels of oxidative stress in patient fibroblasts compared with controls. (**E**) JC-1 staining demonstrates altered mitochondrial membrane potential in patient fibroblasts, shown by the red/green fluorescence intensity ratio. (**F**) Representative gel image of *XBP1* splicing via quantitative reverse transcriptase PCR (RT-qPCR) (top image) and densitometric analysis (bottom left graph) shows elevated levels of spliced *XBP1* (s*XBP1*) in patient fibroblasts compared with controls, confirming UPR activation. Fold change (RT-qPCR) in *XBP1* expression confirms the increase in the spliced form of *XBP1* in patients (bottom right graph). (**G**) Western blot analysis of UPR markers (representative gel image on top) reveals increased abundance of CHOP (bottom left graph) and p53 (bottom right graph) in patient fibroblasts, with quantification normalized to GAPDH. The results are from 3–4 independent experiments and are expressed as mean ± SEM. Statistical significance was determined using the unpaired 2-tailed *t* test and ANOVA (*P ≤ 0.05, **P ≤ 0.001, ***P ≤ 0.0001, ****P ≤ 0.00001).

NO plays a protective role in cells by triggering the activation of different kinases in canonical pathways (23). These include ERK activation, CREB, and AKT, which have notable consequences for neuronal development and survival (23). Intending to identify the impact of decreased NO levels on fibroblasts, we cultured patient and control fibroblasts. We analyzed the DNA fragmentation as well as phosphorylation of AKT, ERK, CREB, and BAD proteins through Western blotting. Terminal deoxynucleotidyl transferase–mediated dUTP nick end labeling (TUNEL) assay revealed that fibroblasts with *CPD* mutations had a larger proportion of cells undergoing apoptosis (Figure 3A and Supplemental Figure 6D). In addition, the patients’ fibroblasts showed significantly higher proportions of cells positive for the apoptosis markers annexin V and propidium iodide (PI) as compared with controls (Figure 3, B and C, and Supplemental Figure 6E). However, when patients’ cells were supplemented with arginine, there was a reduction in apoptosis (*P* < 0.05) among these cells (Figure 3C, Supplemental Figure 6E, and Supplemental Figure 7). Since lysine is also an essential amino acid and is known for its role in cell growth and survival processes, we also tested the effect of lysine in reducing apoptosis in patient fibroblasts; results suggest that it was much less effective or ineffective when administered alone or in combination with arginine (Supplemental Figure 7). Lysine and arginine may interact negatively as they compete for the same transporter (CAT) to enter the same cell. Arginine is well known for its role in metabolic stress adaptation by different cells (24), and lysine is mainly involved in protein and collagen synthesis, and histone modifications (25).

We were unable to identify a significant change in canonical apoptotic protein levels (AKT/p-AKT, CREB/p-CREB, BAD/p-BAD, and caspases) in patient fibroblasts compared with controls (Supplemental Figure 8, A–D). This led us to investigate alternative apoptotic pathways. As mentioned earlier, NO deficiency can have an impact on oxidative stress, mitochondrial dysfunction, ER stress, and/or p53 activation related to apoptosis (17, 18). We therefore tested fibroblasts for oxidative stress by measuring the relative fluorescence intensity of cells after loading with 2′,7′-dichlorodihydrofluorescein diacetate (H_2_DCFDA). The patients’ cells showed significantly increased oxidative stress (Figure 3D and Supplemental Figure 6F) and lower ratios of red/green fluorescence intensity, indicating compromised mitochondrial function (Figure 3E and Supplemental Figure 6G). Oxidative stress and mitochondrial dysfunction are expected to lead to ER stress and p53 activation. As shown in Figure 3, F and G, and Supplemental Figure 6, H and I, spliced *XBP1* (s*XBP1*) and CHOP, markers of ER stress/unfolded protein response (UPR), as well as p53 levels for oxidative stress, were increased in patients’ fibroblasts.

### Silencing of Cpd leads to apoptosis in murine cochlear cells.

**Figure 4:**
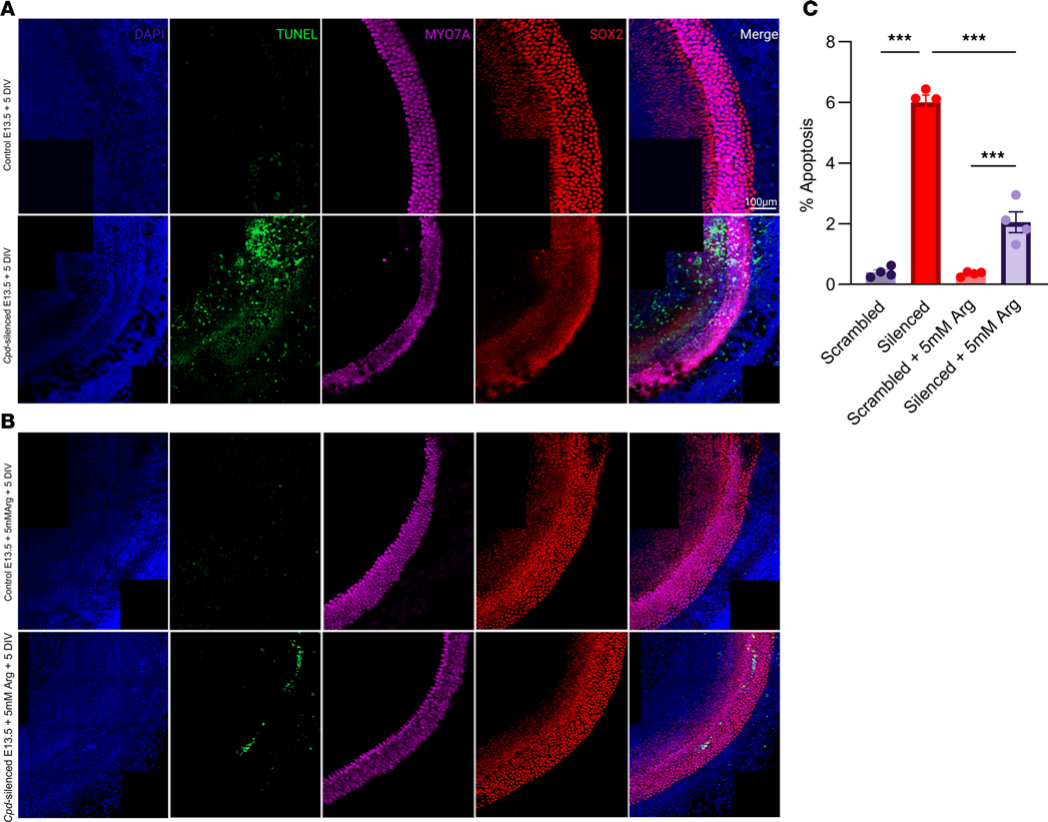
Silencing *Cpd* in the mouse cochlea enhances apoptosis rescued by arginine supplementation. (**A**) Representative confocal images of mouse cochlear explants (E13.5 + 5 days in vitro, DIV) showing increased apoptosis following *Cpd* silencing (infected at MOI 2 × 10^6^), as indicated by TUNEL staining (green). MYO7A (magenta) marks hair cells, SOX2 (red) marks supporting or progenitor cells, and DAPI (blue) labels nuclei. (**B**) Arginine supplementation (5 mM) reduces TUNEL-positive apoptotic cells in *Cpd*-silenced explants. (**C**) Quantification of apoptosis (percentage TUNEL-positive cells per total nuclei). *Cpd* silencing significantly increased apoptosis, which was partially rescued by arginine treatment. Data are presented as mean ± SEM from *n* = 4 explants per group from 2 different litters. No signal was artificially added or removed during the quantification. ***P < 0.001 by 1-way ANOVA with Tukey’s multiple-comparison test. Scale bar: 100 μm.

Since *Cpd* expression was localized in the inner ear, we analyzed the effect of silencing *Cpd* in mouse cochlear explants. The expression of *Cpd* was reduced in cochlea transfected with scrambled (control) and *Cpd* siRNA lentiviruses at a multiplicity of infection (MOI) of 2 × 10^6^, compared with controls (Supplemental Figure 9). We analyzed TUNEL staining signals across apical, mid, and basal regions of the cochlea. Images were acquired at ×63 using an oil immersion lens, and apoptotic cells were counted using ImageJ. The apoptosis was more intense in *Cpd*-silenced explants compared with controls (*P* < 0.001), indicating that sensory cells in the cochlea were undergoing apoptosis due to reduction in *Cpd* expression (Figure 4, A and C). The supplementation of arginine for 24 hours was effective in reducing the apoptosis in the sensory epithelium of the cochlea (Figure 4, B and C). These results suggest that the timely administration of arginine may be beneficial in treating loss of sensory epithelium in the cochlea.

### Loss of silver impairs auditory processing and disrupts scolopidia organization.

**Figure 5:**
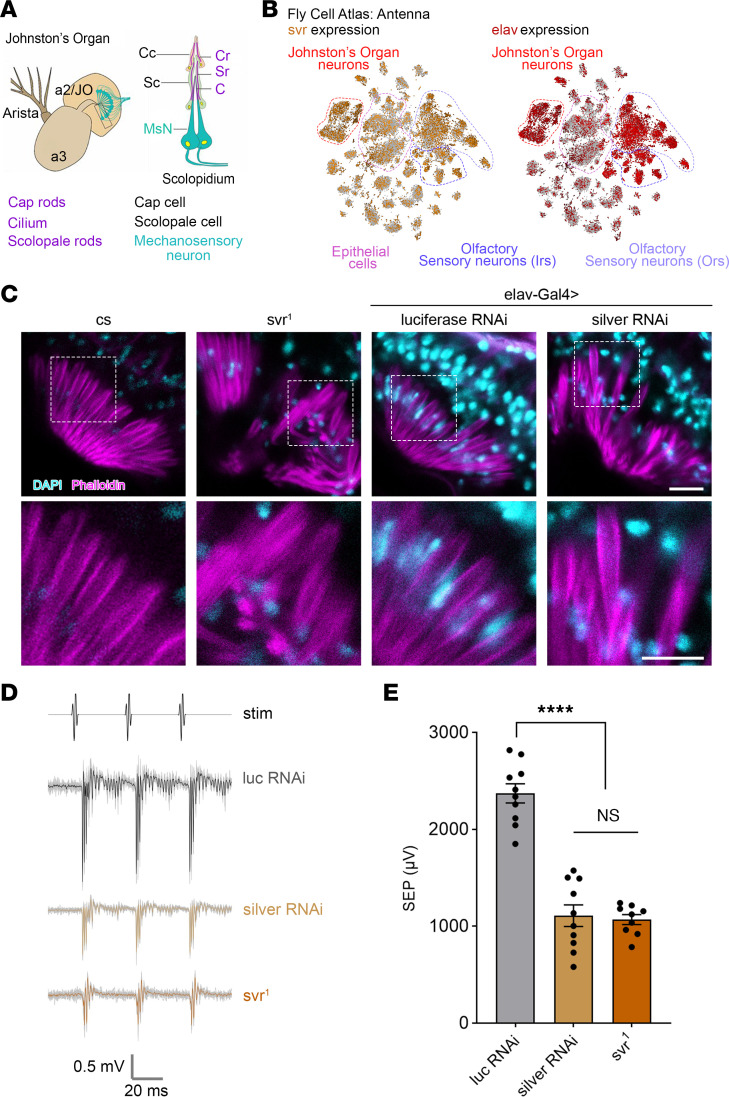
Loss of *silver* results in impaired morphology and function of Johnston’s organ. (**A**) Left: Schematic representation of *Drosophila* antenna. Mechanosensory neurons (labeled in cyan) are suspended within Johnston’s organ, located in A2 segment. Right: Schematic showing the organization of one scolopidium, including scolopale rods and actin bundles in the cilium, labeled in magenta (boxed areas in **C**). Adapted with permission from *Proceedings of the National Academy of Sciences of the United States of America* (28). (**B**) t-Distributed stochastic neighbor embedding (t-SNE) plot showing *svr* (gold) and *elav* (red) expression in the antenna using SCope: Fly Cell Atlas dataset (31). (**C**) Confocal micrographs showing phalloidin labeling of scolopale rods and actin bundles in the cilium (magenta), and nuclei labeled with DAPI (cyan), of *cs* or *luciferase-RNAi* control compared with *svr^1^* and *silver-RNAi*. Boxed areas highlight zoomed-in regions of disorganized actin bundles. Scale bars: 10 μm. (**D**) Representative sound-evoked potential (SEP) traces recorded from antennae in response to presentation of simulated pulse song (stim). For each trace, the responses of 10 consecutive stimuli are shown in light gray, with the average of these 10 traces shown in the color representative of each genotype. (**E**) SEP amplitudes are plotted with each point representing a different antenna. Bars represent means, error bars represent SEM. Statistical significance was determined using ANOVA (*P* < 0.0001) and Tukey’s post hoc comparisons (****P < 0.0001).

The *Drosophila* ortholog of CPD, encoded by the *silver* gene (*svr*), is highly conserved and serves as a robust model for CPD dysfunction with a DIOPT (Drosophila RNAi Screening Center Integrative Ortholog Prediction Tool; https://www.flyrnai.org/tools/diopt/web/api) score of 12/16 (26). The *silver* gene is alternatively spliced to produce long transmembrane forms with 3 CP-like domains and short soluble forms with a single CP domain (27). Recent studies have highlighted the molecular and genetic conservation of the mechanosensory transduction in flies and vertebrates (28–30). Analysis of scRNA-Seq of the adult *Drosophila* antenna obtained from Fly Cell Atlas (31) with SCope visualization confirmed that *svr* was highly expressed in the antenna, including Johnston’s organ (JO), the *Drosophila* hearing organ (Figure 5, A and B). JO is the component of the auditory system required for sensing gravity, wind flow, and near-field sound (32).

Distinct classes of *svr* mutants have been described. Global loss-of-function alleles that reduce all CPD isoforms result in embryonic or larval lethality. In contrast, mutants that specifically affect long forms, such as *svr^1^* mutant (a 3 bp deletion in exon 6, causing loss of a conserved leucine residue in the second CP-like domain and consequent loss of enzyme activity and protein misfolding), reduce the abundance of the long form and the activity of CPD (27). To further model CPD loss, we performed pan-neuronal knockdown (RNAi) of *svr* using *elav-GAL4*, which is significantly expressed in the antenna, including the JO neurons (Figure 5B).

JO contains approximately 200 scolopidia (Figure 5A), the fundamental mechanosensory units of hearing analogous to vertebrate hair cells (33). To assess the effect of loss of function of *svr* on JO, we assessed morphology of scolopidia using phalloidin staining. Both *svr^1^* mutants and pan-neuronal *silver*-*RNAi* flies exhibited disorganized actin bundles in JO when compared with their respective controls, indicating deterioration of auditory organ structure (Figure 5C).

To assess whether loss of *svr* affects auditory function, we performed electrophysiology recordings of sound-evoked potentials (SEPs) from the antennal nerve. Control flies (*luciferase-RNAi*) exhibited robust SEP amplitudes, while *silver-RNAi* or *svr^1^* mutants displayed an approximately 50% reduction in SEP amplitudes (Figure 5, D and E), indicating significant impairment of auditory transduction. Collectively, these findings demonstrate that CPD deficiency caused by *svr* mutations or knockdown leads to both structural and functional impairment of the auditory system.

### Loss of silver causes deficits in negative geotactic behavior in Drosophila.

**Figure 6:**
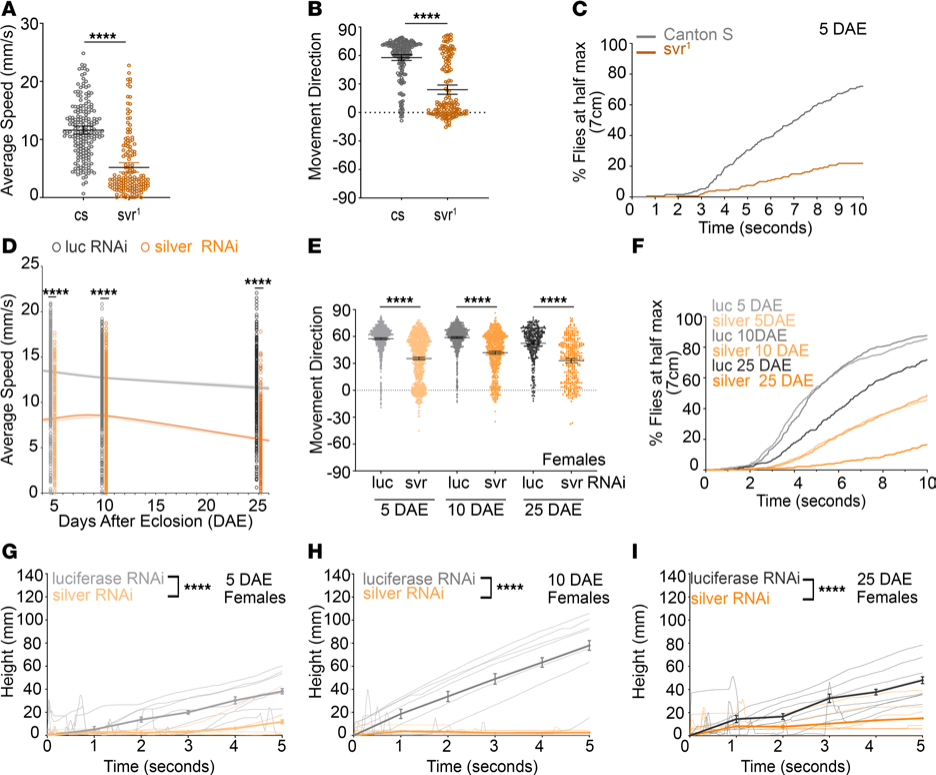
*Silver* loss of function results in impaired negative geotaxis behavior. (**A**–**C**) Negative geotaxis analysis of *canton-s* (*cs*) (gray) control or *svr^1^* (gold) in 5-DAE female flies. Assessment of average speed (**A**), movement direction (**B**), and percentage of flies that reached 7 cm (half maximum) in 10 seconds for *cs* control (*n* = 190) or *svr^1^* (*n* = 154) (**C**). (**D**–**I**) Negative geotaxis analysis of pan-neuronal knockdown of *luciferase* control (gray) or *silver* (orange) in female flies. Assessment of average speed (**D**), movement direction (**E**), percentage of flies that reached 7 cm in 10 seconds (**F**), climbing distance at 5 DAE for *luciferase* control (*n* = 770) or *silver* (*n* = 1,048) (**G**), climbing distance at 10 DAE for *luciferase* control (*n* = 740) or *silver* (*n* = 775) (**H**), and climbing distance of 25 DAE for *luciferase* control (*n* = 350) or *silver* (*n* = 350) (**I**). Data are presented as mean ± 95% CI. ****P < 0.0001.

To determine whether *CPD* deficiency impacts broader JO-mediated behaviors, we assessed negative geotactic behavior, a sensitive measure of gravity sensing and motor coordination reliant on intact auditory/neurosensory circuits (26, 34). Therefore, while negative geotaxis is not solely a measure of auditory processing, it relies on the function of the antenna and JO, which are critical for sound detection. Compared with wild-type *canton-s* (*cs*) controls, *svr^1^* showed significantly reduced speed (Figure 6A), movement direction (Figure 6B), and percentage of flies to reach a determined distance (7 cm) in 10 seconds (Figure 6C). Additionally, longitudinal analysis of *silver-RNAi* revealed sustained deficits in speed and movement direction across ages (5, 10, and 25 days after eclosion [DAE]) in both females (Figure 6, D and E) and males (Supplemental Figure 10, A and B), along with reduced percentage to reach 7 cm in 10 seconds (Figure 6F). Last, we also measured the distance climbed in the first 5 seconds, and the kymograph showed a significant reduction in climbing rate for *silver-RNAi*–knockdown female (Figure 6, G–I) and male flies when compared with control at 5, 10, and 25 DAE, respectively (Supplemental Figure 10, C–E). This suggests a delayed response in gravity sensing for the *silver-RNAi* flies when compared with the controls. Taken together, the results suggest that loss of *silver* by mutant deletion or pan-neuronal knockdown causes impaired negative geotactic behavior, and, when combined with reduced auditory processing and impaired morphology, suggest impaired function of the *Drosophila* hearing organ.

### Feeding with l-arginine or sildenafil improves negative geotactic behavior in Drosophila model of CPD deficiency.

**Figure 7:**
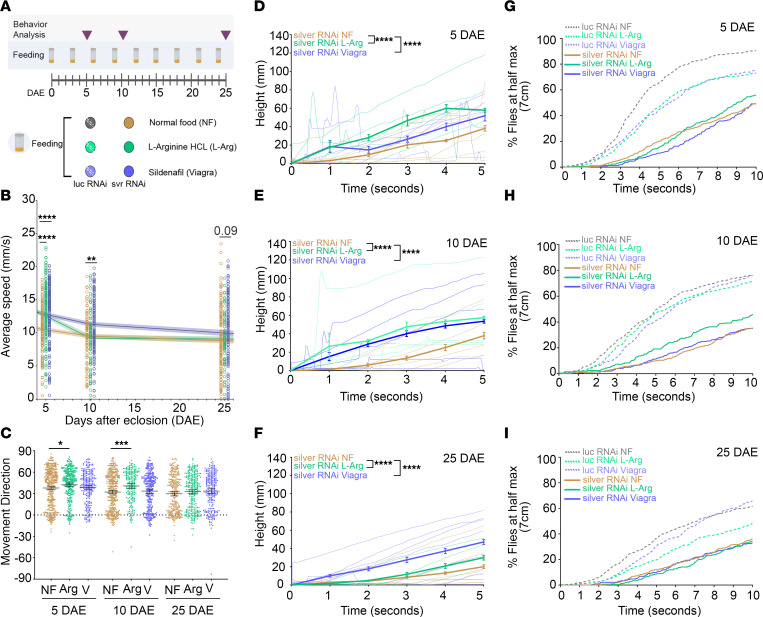
Feeding with l-arginine or sildenafil improves negative geotaxis behavior in flies with pan-neuronal *silver* knockdown. (**A**) Feeding paradigm for normal food (NF), l-arginine (L-Arg), and sildenafil (Viagra). (**B**–**I**) Negative geotaxis behavior for females with pan-neuronal knockdown of *silver* (*elav^C155-GAL4^* > *UAS-silver-RNAi*) fed with either NF (gold) (*n* = 140), L-Arg (green) (*n* = 180), or Viagra (blue) (*n* = 180). Assessment of average speed (**B**), movement direction (**C**), climbing distance at 5 DAE (**D**), 10 DAE (**E**), and 25 DAE (**F**), and percentage of *luciferase* (dashed lines) or *silver* (solid lines) flies to reach 7 cm in 10 seconds for 5 DAE (**G**), 10 DAE (**H**), and 25 DAE (**I**). Data are presented as mean ± 95% CI. **P < 0.01, ***P < 0.001, ****P < 0.0001.

To test whether modulating the NO pathway could rescue deficits caused by CPD deficiency, we examined the effects of l-arginine and sildenafil, a cGMP enhancer, in flies with pan-neuronal knockdown of *silver* by assessing the negative geotactic function of the flies after drug feeding at previously analyzed time points (Figure 7A). Both l-arginine and sildenafil significantly improved the average speed for *silver-RNAi* at 5 DAE, and sildenafil continued to show improvement at 10 DAE (Figure 7B). Moreover, l-arginine significantly improved movement direction at 5 and 10 DAE when compared with normal food, while sildenafil showed minor improvements (Figure 7C). Feeding with both drugs significantly improved distance climbed in the first 5 seconds at all time points, showing significant improvement of gravity sensing (Figure 7, D–F). Lastly, the percentage of flies that were able to reach 7 cm in 10 seconds also improved with l-arginine supplementation (Figure 7, G–I). Unexpectedly, l-arginine or sildenafil feeding in control flies occasionally caused mild behavioral toxicity, evidenced by decreased performance (Supplemental Figure 11), suggesting context-dependent effects.

Overall, these findings demonstrate that loss of CPD/silver impairs the structure and function of the *Drosophila* auditory system and associated gravity-sensing behaviors, but that these deficits can be partially ameliorated by pharmacological modulation of the NO/cGMP pathway. This suggests the beneficial effects of modulating NO pathways in enhancing the integrity and output of sensory-motor neurocircuitry in the context of CPD loss of function and highlights the translational potential of targeting this signaling cascade in conditions of CPD-deficient hearing loss.

## Discussion

CPD belongs to a family of metallocarboxypeptidases, which perform diverse physiological functions, ranging from the digestion of food to the biosynthesis of neuropeptides (35). It is a single-transmembrane protein harboring 3 catalytic carboxypeptidase domains, each of which requires a Zn^2+^ divalent ion as a cofactor (36, 37).

CPD’s role in human health has been suggested in a few studies related to lupus erythematosus (38) and cancer (39); however, to our knowledge, a hereditary disorder caused by CPD dysfunction has not been reported until this study. There are several known mutations in *CPD* in the ortholog of *Drosophila* that are collectively known as the *silver*, or *svr*, mutants. Along with the viable *svr* mutants there are many mutants reported to be embryonic lethal in the literature (38). Moreover, *Cpd*-knockout mice do not survive (International Mouse Phenotyping Consortium; https://www.mousephenotype.org/). It is possible that complete loss of CPD function is not compatible with life in humans. In our study, we report missense *CPD* variants exclusively located within CP domain 2 that are predicted to disturb the catalytic activity of the domain. Our results show that catalytic inactivity leads to the reduction of available arginine in cells, which feeds into the NO signaling pathway. It has been shown that accumulation of NO in the cochlea protects cells from noise-induced HL (39, 40). In addition, *Prkg1* and *Pde5*, the two essential molecules of the cGMP signaling cascade, are highly expressed in outer and inner hair cells. The cGMP/*Prkg1* signaling had a protective role against cell damage by noise-induced HL in mice, exemplified by the administration of a PDE5 inhibitor to enhance cGMP (40).

While NO signaling is widespread in mammalian physiology, the selective impact of *CPD* deficiency on the auditory system is striking. The cochlea lacks regenerative capacity and relies on tightly regulated signaling mechanisms for cochlear cell survival and function. In contrast, other tissues may be protected by functional redundancy from alternative enzymes or compensatory/repair metabolic pathways (41, 42). Thus, the auditory system may represent a unique site affected by subtle perturbations in arginine metabolism.

Mutations in a few genes have been shown to initiate ER stress and unfolded protein response (UPR), leading to HL. *GJB2* or *GJB6* mutant proteins can become trapped in the ER, preventing these gap junction proteins from transferring to the plasma membrane (43, 44). Similarly, levels of the anti-apoptotic protein Bip were elevated in the cells overexpressing pathogenic *GJB3* variants (45). *CDH23*, *USH1C*, and *MYO7A* are involved in the preassembly of the Usher protein complex at the ER in zebrafish hair cells, and defects in any one of the 3 proteins can induce ER stress, triggering apoptosis (46). Very recently, germline mutations in *ATF6*, a key regulator of UPR, have been reported to cause SNHL in humans and mice (47). Our study links NO deficiency with metabolic stress that induces ER stress and the UPR pathway, leading to HL.

We show that this type of HL is potentially treatable if the cellular stress is reversed by the supplementation of arginine. The addition of l-arginine to the medium rectifies NO and cGMP levels and apoptosis in patients’ fibroblasts. Moreover, treating *Drosophila* CPD deficiency models with l-arginine or sildenafil improves their gravity sensing, as shown by enhanced negative geotactic behavior, a process that requires the hearing organ JO, highlighting the beneficial effects of modulating NO pathways to improve the function of sensory-motor neurocircuitry.

This study combines human genetics with mechanistic validation in patient-derived fibroblasts, cochlear explants, and *Drosophila*, providing a comprehensive view of *CPD* deficiency as a cause of HL. The use of cross-species modeling and demonstration of rescue via l-arginine or sildenafil highlights the therapeutic potential of targeting the NO pathway. Limitations include the lack of a mammalian in vivo model demonstrating auditory behavior and the relatively small number of affected individuals with biallelic variants. However, heterozygous loss-of-function variants in *CPD* may be a more common risk factor for SNHL with increasing age. Future work will focus on developing cellular and vertebrate models for in vivo auditory testing and evaluating combinatorial therapies involving metabolic supplementation and gene delivery.

## Methods

### Sex as a biological variable.

Our study included both male and female patients. We used both male and female mice in the experiments. Sex was not a biological variable.

### Enrollment of participants.

After enrollment, participants underwent a comprehensive clinical assessment to evaluate HL and related findings. Audiological examinations were performed in a soundproof room following standard procedures (48). We collected detailed medical and family histories; conducted thorough physical examinations, including neurological and ophthalmological assessments; and obtained laboratory and radiological tests to identify the cause of HL.

### Molecular analysis.

After excluding *GJB2* variants by Sanger sequencing, we initially performed exome sequencing in the proband of each family (IV:3, II:1, and II:1 in families 1, 2, and 3, respectively). Subsequently, for a comprehensive evaluation of coding and non-coding variants, we performed genome sequencing in the proband of family 1. Details of bioinformatic analyses and variant prioritization are provided in Supplemental Methods.

### Enrichment and prioritization of protein-altering variants in CPD from a large cohort.

We queried genome sequencing data from 65,523 participants in 100KGP with rare diseases or cancer, aligned to the GRCh38 build. Participants were searched for inclusion under the “hearing and ear disorders” category within their normalized disease group. Additionally, we searched for participants with any HL phenotype referenced in the free text of any Human Phenotype Ontology or ICD-10 term. Individuals not classified by 100KGP under “hearing and ear disorders” but with identified HL phenotypes were grouped accordingly.

This process identified a total of 3,802 unrelated probands with HL. Controls consisted of 27,503 participants recruited under other disease categories without any associated HL phenotype. Relatives were excluded from the analysis. Cases and controls were matched for age, sex, and ancestry.

Briefly, 4 categories of variants in the *CPD* gene were analyzed: (a) Protein-altering variants included those classified by functional annotations such as frameshift variants, stop-gained variants, and missense variants. No filtering was applied for minor allele frequency or in silico prediction tools. (b) LoF variants included predicted damaging variants such as stop-gained, splice-acceptor, and splice-donor variants, all of which were considered rare and damaging. (c) Prioritized missense variants were restricted to those with a minor allele frequency less than 0.001 and either a Combined Annotation Dependent Depletion score ≥ 25 or an AlphaMissense (https://alphamissense.hegelab.org/) score indicating probable damage or ambiguity (≥0.34). Finally, (d) combined variants included those meeting the criteria for both prioritized missense and LoF categories.

For each variant category, alternate allele counts and total allele numbers were calculated for cases and controls. Frequencies were expressed as proportions of alternate alleles to total alleles. ORs were calculated to evaluate the association between variant presence and HL, along with 95% confidence intervals (CIs). Fisher’s exact test was used to compute *P* values, and statistical significance was defined as *P* ≤ 0.05. Analyses were performed using standard statistical software.

### Protein modeling.

Homology modeling of human CP domain 2 (residues 501–875) of the wild-type CPD and the variant p.(Met563Arg) (family 1)/p.(Arg833His) (family 2)/p.(Gln791Arg) (family 3) triplet in complex with the peptidomimetic inhibitor GEMSA was performed using MODELLER (https://salilab.org/modeller/) (49). The crystal structure of duck CP domain 2 bound to GEMSA (Protein Data Bank ID: 1H8L) was used as a template having sequence identity close to 85%. A total of 100 atomic models were calculated, and the best structure with the lowest energy, determined by the MODELLER Objective Function, was selected for further analysis. The structural models were rendered using RIBBONS (50).

### Localization of CPD in mouse inner ear and antibody validation.

To assess the localization of CPD, tympanic bullae containing cochlea were dissected from P0 C57BL/6 wild-type mice. They were locally perfused with 4% paraformaldehyde through the round and oval windows and kept at 4°C overnight. Next day the cochleae were rinsed in 1× phosphate-buffered saline (PBS) and permeabilized with 0.25% Triton X-100. Blocking was done in 5% bovine serum albumin (BSA) for 1 hour at room temperature. Incubation was done overnight at 4°C with the following primary antibodies: rabbit anti-CPD polyclonal antibody (Invitrogen, catalog CPD PA5-103707), mouse anti-MYO7A monoclonal antibody (MYO7A 138-1; Developmental Studies Hybridoma Bank, University of Iowa, Iowa City, Iowa, USA), and a chicken anti-neurofilament polyclonal antibody (MilliporeSigma, catalog AB5539). Nuclei were stained with DAPI, and images were captured with Zeiss LSM 710 or Zeiss LSM 980 with AiryScan 2 confocal microscope (Carl Zeiss).

The sensitivity of rabbit anti-CPD polyclonal antibody was tested and validated in CRISPR/Cas9–mediated CPD knockout in HEK293 cells (ATCC, CRL-1573). *CPD-*knockout lines clone 5, clone 14, and clone 55 were prepared using 5′-GAATCACAAACGGCGCACAT-3′ and 5′-CGGCGCACATTGGTATGATG-3′ guides and validated as previously described (51). The Sanger-validated clones (Supplemental Figure 12) were grown in Dulbecco’s modified Eagle medium (DMEM) supplemented with Normocin (InvivoGen, catalog ant-nr-05) and 10% FBS (Thermo Fisher Scientific, catalog A5256701) at 37°C, 5% CO_2_, and 95% humidity. Upon reaching 70% confluence, cells were fixed with 4% paraformaldehyde in PBS for 30 minutes at room temperature, followed by permeabilization with 0.3% Triton X-100 in PBS for 10 minutes, and then stained with rabbit anti-CPD primary antibody overnight. The following day, cells were incubated with anti-rabbit Alexa Fluor 488 secondary antibody (Invitrogen, catalog A-11008) and DAPI. Images were captured using a Zeiss LSM 980 AiryScan 2 confocal microscope.

Additionally, cells were transfected with (*CPD* pcDNA3.1+/C-(K)DYK[(NM_001304.4) GenScript]) expression plasmid using the jetPRIME transfection reagent (Polyplus). After 72 hours, cells were fixed with 4% paraformaldehyde in PBS for 30 minutes at room temperature, permeabilized with 0.5% Triton X-100 in PBS for 10 minutes, and then costained with mouse monoclonal FLAG tag (Cell Signaling Technology, catalog 8146S) and rabbit polyclonal CPD primary antibodies overnight. The following day, cells were incubated with anti-mouse Alexa Fluor 488 (Invitrogen, catalog A-11001) and anti-rabbit Alexa Fluor 555 (Invitrogen, catalog A-21428) secondary antibodies, respectively. Subsequently, the cells were counterstained with DAPI and mounted in Prolong Glass antifade solution (Invitrogen). Images were acquired using a ×63 objective on a Zeiss LSM 980 AiryScan 2 confocal microscope.

### CPD enzyme activity.

To measure the C-terminal cleavage activity of the carboxypeptidase D, the enzyme was incubated with the fluorescent substrate dansyl-Phe-Ala-Arg (Bachem, catalog 4028360.0005). Briefly, HEK293 cells were grown and maintained in DMEM supplemented with 10% FBS and 1× antibiotic-antimycotic solution at 37°C, 5% CO_2_, and 95% humidity. Upon 70%–80% confluence, the cells were transfected with the *CPD* expression plasmid (GeneCopeia, EX-OHU10876D) using jetPRIME transfection reagent. The mutated constructs (CPD^c.1688T>G^, CPD^c.2372A>G^, CPD^c.2498G>A^) were generated using QuikChange Lightning Site-Directed Mutagenesis kit (Agilent Technologies, catalog 210518) (Supplemental Table 3). After 72 hours of transfection, cells were harvested and lysed in 100 mM Tris-acetate (pH 6.4) containing 100 mM NaCl. One hundred microliters of supernatant in a microcentrifuge tube was incubated with 0.2 mM of substrate for 60 minutes at 37°C. The reaction was stopped by addition of 50 μL of 500 mM HCl and 1 mL of chloroform. Tubes were mixed and centrifuged for 2 minutes at 300*g*. When centrifugation was complete, the bottom phase was separated into a fresh tube and dried overnight at room temperature in the dark. Dried samples were resuspended in 200 μL of PBS containing 0.1% Triton X-100. Fluorescence was measured at 495/350 nm using a 96-well microplate reader (Synergy H1m Monochromator-Based Multi-Mode Microplate Reader) with Gen5 2.0 data analysis software (both from Agilent).

The impaired enzyme activity was also confirmed by peptidomics analysis at Creative Proteomics.

### Fibroblast studies and effects of l-arginine supplementation.

Fibroblasts from 6 controls and 4 patients were plated in 150 × 20 mm cell culture Petri dishes (Thermo Fisher Scientific, catalog 150468) using DMEM for stable isotope labeling by amino acids in cell culture (SILAC) (Thermo Fisher Scientific, catalog A33822) supplemented with 10% dialyzed FBS (Thermo Fisher Scientific, catalog 26400044) and 1× antibiotic-antimycotic solution. The medium was changed each alternate day, and cells were kept growing until 80% confluent. The cells were harvested in 100 μL arginine assay buffer provided with the Arginine Assay Kit (Abcam, catalog AB252892). Cells were homogenized and centrifuged at 10,000*g* for 10 minutes at 4°C. Supernatant was prepared, and reagents were added as described by the manufacturer. The final incubation was at 37°C for 1 hour. Fluorescence was measured at 535/587 nm on a microplate reader in endpoint mode.

Similarly, for measurement of intracellular lysine, cells were collected in 100 μL lysine assay buffer provided with the Lysine Assay Kit (Cell Biolabs Inc., catalog MET-130). Cells were lysed and centrifuged as above, and fresh lysates were used for the experiment. Standards were freshly prepared, and lysates from the respective groups were incubated with the colorimetric probe as described by the manufacturer. Readings were measured with a spectrophotometric plate reader at 550 nm.

For NO and cGMP measurements, upon 80% confluence, the medium was supplemented with 6 mM l-arginine (Thermo Fisher Scientific, catalog A14730.22) for 48 hours. Cells were harvested after 48 hours and homogenized in RIPA buffer (Thermo Fisher Scientific, catalog 89900) mixed with 1× Halt protease and phosphatase inhibitor cocktail (Thermo Fisher Scientific, catalog 78441). Each sample was then centrifuged at 12,000*g* for 15 minutes at 4°C. The supernatant was collected, protein estimation was conducted via bicinchoninic acid assay (Sigma-Aldrich, catalog B9643), and absorbance was measured at 562 nm using a microplate reader. For NO measurement, incubation was done for 10 minutes at 60°C, and absorbance was measured at 540 nm according to an NO assay kit (Abcam, catalog ab272517). For cGMP levels, cells were harvested after 48 hours in 0.1 M HCl and hydrolyzed to obtain total cell lysates. The absorbance was measured at 412 nm in a 96-well ELISA plate (Cayman, catalog 581021). Raw data were analyzed using the provided web tool (https://www.myassays.com/cyclic-gmp-acetylated.assay).

### Apoptosis detection assays.

Apoptosis was observed with TUNEL staining of patients’ fibroblasts. Approximately 5 × 10^4^ cells were plated on glass coverslips, and TUNEL assay was performed using the 1-step TUNEL in situ apoptosis kit (Biotium, catalog 30063) according to the manufacturer’s protocol. Images were quantified using ImageJ software (NIH). The images were split into respective channels, and thresholding was done to highlight only the cells. The count particle function was used to get the number of cells detected in each channel. Percentage apoptotic cells was calculated and plotted using GraphPad Prism software (version 10.0.2).

Fibroblasts from patients and control samples were also tested for apoptosis using flow cytometry. Cells were collected, washed with PBS, and stained with an Annexin V–FITC/PI detection kit (BioLegend, catalog 640914). Stained cells were analyzed using a BD FACSAria IIu Flow Cytometer, and data were processed using FlowJo software. Gating details are provided in Supplemental Figure 13.

Additionally, we performed Western blot analysis to investigate the role of conventional proteins for apoptosis in patients and control fibroblasts. Cells were harvested in RIPA lysis buffer, and the extracted proteins were resolved through a 4%–20% Tris-glycine gradient gel and then transferred to a 0.22 μm PVDF membrane. The membrane was then blocked in 5% BSA for 1 hour and incubated overnight at 4°C with primary antibodies ERK1/2 (Cell Signaling Technology, catalog 9532S), p-ERK1/2 (Cell Signaling Technology, catalog 5625S), AKT (Cell Signaling Technology, catalog 9272S), p-AKT (Cell Signaling Technology, catalog 4060S), BAD (Cell Signaling Technology, catalog 9239S), p-BAD (Cell Signaling Technology, catalog 4366S/9291S), CREB (Cell Signaling Technology, catalog 4820S), p-CREB (Cell Signaling Technology, catalog 9198S), and caspase-3 (Cell Signaling Technology, catalog 14220S) each diluted at 1:1,000 in 5% BSA plus TBST (TBS with 0.5% Tween). The next day, the membrane was washed and incubated with anti-rabbit or anti-mouse peroxidase-conjugated secondary antibody (1:3,000) and developed using West Pico Super-Signal ECL substrate (Thermo Fisher Scientific, 37069). Finally, visualization was performed using FluorChemE (ProteinSimple). Quantification was done using ImageJ software.

Oxidative stress in control and patient fibroblasts was measured with H_2_DCFDA (Thermo Fisher Scientific, catalog D399) fluorometric assay. Approximately 7,000 cells were seeded in 96-well cell culture plates. After 24 hours, the cells were loaded with 100 μL of 10 μM H_2_DCFDA and incubated for 30–40 minutes at 37°C. Subsequently, cells were washed, and volume was replaced with pre-warmed HBSS. The plate was read at excitation/emission 485/535 as discussed earlier.

Mitochondrial membrane potential is a good indicator of the healthy state of cells. To observe the mitochondrial dysfunction in control and patients’ fibroblasts, the cells were maintained as discussed above. A 1:1,000 working solution of JC-1 (Cell Signaling Technology, catalog 92891), a ratio-metric fluorescent reporter of mitochondrial membrane potential, was prepared according to the manufacturer’s protocol to stain the cells after incubation for 30 minutes. Cells were washed and passed through a cell strainer before the FACS analysis. A FACSAria IIu Flow Cytometer with lasers (488 and 561 nm) and filters (525/50 and 585/42 nm) was used, and data were processed using FlowJo software. Details of the gating strategy are given in Supplemental Figure 13.

### ER stress marker quantification.

Spliced *XBP1* (s*XBP1*), associated with pro-homeostatic activity of the UPR and CHOP, correlated with pro-apoptotic activity of the UPR and was tested and quantified in cultured fibroblasts. Briefly, cells were grown as described above. RNA was extracted when cells reached 70%–80% confluence. Spliced and unspliced forms of *XBP1* were measured by semiquantitative PCR and quantitative PCR (qPCR) and quantified against *GAPDH*. Densitometric analysis was performed using ImageJ, and intensities were recorded for further statistical analysis.

The lysates from the fibroblasts were prepared for Western blot analysis as described in the previous section. The membranes were incubated with anti–rabbit CHOP primary antibody (Proteintech, catalog 15204-1-AP; 1:1,500) overnight, and blots were developed after staining with the respective secondary antibody. Quantification was performed using ImageJ analysis software, and plots were generated for relative levels of CHOP in both controls and patients, normalized to GAPDH.

### Organotypic cultures of mouse cochlea and Cpd silencing.

C57BL/6 mice were time-mated to obtain E13.5 embryos. Cochleae from embryos confirming E13.5 staging criteria were used for culture. Dissection was performed as previously described (52), and 4 cochleae were placed on each Millicell cell culture insert. *Cpd* RNAi lentiviral system (sc-142542-V, sc-108080 [control], and sc-142542-PR) was purchased from Santa Cruz Biotechnology Inc. After 3 days of culture, cochleae were transduced with viral particles at 1 × 10^6^, 2 × 10^6^, and 4 × 10^6^ MOI. The medium was changed the following day, and cochlea was cultured until E18.5. The efficiency of transduction was measured with qPCR, and fold change in *Cpd* expression was determined. To quantify the apoptosis in cochlear cells, the tissue was harvested at E18.5 and fixed, and a 1-step TUNEL assay was performed. MYO7A, SOX2, and CPD antibodies were used to detect any differences in lateral and medial domains of silenced and wild-type specimens. Imaging was performed using a Zeiss LSM 980 microscope equipped with a ×63/1.4 NA oil immersion objective. *Z*-stacks were acquired with tiling using a zoom factor of ×1.2 and a frame size of 2,048 × 2,048 pixels. All images were captured with consistent laser power, gain, and offset settings. Maximum-intensity projections were generated from *Z*-stacks using ImageJ. To quantify apoptosis, 3 regions of interest (ROIs) from the apical mid and basal turns in each image, encompassing the cochlear sensory epithelium, were manually selected. The Threshold function was applied to the green channel to identify TUNEL-positive cells using consistent settings across all images. The number of apoptotic cells was quantified using the Analyze Particles tool with size and circularity parameters optimized to exclude background noise. The number of TUNEL-positive cells was normalized to the total number of DAPI-stained nuclei within the same ROI to calculate the percentage of apoptotic cells.

The effect of arginine supplementation to overcome apoptosis in the cochlea was also tested. Briefly, the culture was done as described above; however, the cochleae were kept in a modified medium composed of DMEM for SILAC supplemented with 10% dialyzed FBS, 1× N-2 supplement (Thermo Fisher Scientific, catalog 17502001), 1× B-27 (minus antioxidants) (Thermo Fisher Scientific, catalog 10889038), and 1× penicillin. We tested 3 different concentrations of arginine (3 mM, 5 mM, and 6 mM) to evaluate the effective dose for the rescue of apoptosis in the cochlea (data not shown). After repeated experimentation and analysis, we selected 5 mM arginine as the best dose to be added at E17.5 for 24 hours. The tissue was harvested at E18.5, fixed, and stained for apoptotic and sensory epithelial markers. Quantification and statistical analysis of images from 3 ROIs on each cochlea (*n* = 4) were done using ImageJ and GraphPad Prism.

### Drosophila studies using the automated geotaxis monitoring platform.

*elav^C155^-Gal4*, *svr^1^*, and *UAS-silver-RNAi* strains previously generated and confirmed were obtained from the Bloomington Drosophila Stock Center (Indiana University, Bloomington, Indiana, USA) (53). The fly strains *canton-s* (*cs*) and *UAS-luciferase-RNAi* were used as controls. Behavior assay was performed on an automated behavior monitoring system as previously published (54). Briefly, each behavior cylinder was preloaded with 7 or fewer flies, and the geotaxis of each fly was recorded with a digital camera (ImagingSource LLC, model DMK23U445). The 2-dimensional positions (*x*, *y* coordinates) of individual flies (maximum height, 14 cm) at 33-millisecond resolution (30 fps) were determined and tracked. Finally, the difference in SD of horizontal and vertical positions was used to calculate movement direction. Specifically, for a given fly, movement direction = (SD of *y* coordinates – SD of *x* coordinates)/(SD of *y* coordinates + SD of *x* coordinates). Matlab (Mathworks) was used for analysis.

### Whole mount of Drosophila Johnston’s organ.

Antennae were detached from the heads and fixed in freshly made 4% formaldehyde (in PBS, pH 7.4) with 0.01% PBTx (PBS containing 0.4% vol/vol Triton X-100) for 20 minutes. The antennae were then washed with 0.4% PBTx 3 times for 15 minutes and incubated overnight with Alexa Fluor 546–conjugated phalloidin (Invitrogen, A22283; 1:200) diluted in 0.4% PBTx with 5% normal goat serum at 4°C, followed by DAPI (Invitrogen, D1306; 1:300) staining for 10 minutes. After washing, tissues were mounted with VECTASHIELD Antifade Mounting Medium (Vector Laboratories) and imaged. Slides were imaged using an Olympus FV4000 confocal microscope with a ×60 oil immersion objective lens with ×2.0 zoom, with a scan speed of 2.0 microseconds per pixel and spatial resolution of 2,048 × 2,048 pixels. Images were processed using cellSens FV (Evident Corporation).

### SEPs of Drosophila antenna.

SEPs were captured using a pair of electrolytically sharpened tungsten recording electrodes (55, 56). The recording electrode was inserted between the first and second antennal segments, while the reference electrode was inserted into the head cuticle near the posterior orbital bristle. A computer-generated pulse song was introduced frontally to the fly under near-field conditions. Signals were subtracted and amplified with a differential amplifier (World Precision Instruments, DAM50) and digitized at 10 kHz (National Instruments, USB-6001). Average response values were measured as the maximum – minimum in an averaged trace from 10 consecutive presentations of the described protocol.

### Statistics.

Statistical analysis was performed by application of Tukey’s multiple-comparison test. One-way ANOVA with Dunnett’s or Šidák’s multiple-comparison test or 2-way ANOVA with Tukey’s multiple-comparison test was used to compare multiple groups. *P* ≤ 0.05 was considered statistically significant. All statistical analyses were performed in GraphPad Prism software (version 10.0.2 or 10.0.3).

### Study approval.

The study was approved by the Institutional Review Board at the University of Miami (protocol 20081138, USA) and the Ankara University Medical School Ethics Committee (protocol 012413). All participants (or parents/guardians) provided written informed consent in accordance with the Declaration of Helsinki protocol. Wild-type C57BL/6 mice were bred and maintained at the University of Miami, where all procedures were approved by the Institutional Animal Care and followed the NIH Intramural Research Program policy, “Using Animals in Intramural Research” (revised 2023).

### Data availability.

Genome and exome sequencing data were deposited in the NCBI’s BioProject database: exome sequencing project PRJNA1079783, samples SAMN40082632 (Family_1_IV:3_exome), SAMN40082633 (Family_2_II:1_exome), and SAMN40082634 (Family_3_II:1_exome); genome sequencing project PRJNA1079835, sample SAMN40090193 (Family1_IV:3_Genome). Primary data from the 100KGP database are held in a Secure Research Environment and are available to registered users. Other data generated or analyzed during this study are included in this article and its supplemental material. This article contains supplemental information comprising Supplemental Methods, Figures, and Tables. All supporting data values underlying the figures in this article are provided in the Supporting Data Values file.

## Author contributions

MR and MT conceptualized the study. MR, MFZ, AGL, NOV, CA, AF, NV, MT, NB, S Guo, STY, MKY, VC, MCR, TC, and MAJ collected data. MR, GB, S Guo, NV, DD, AGL, NOV, CA, AF, and FTE performed formal analysis. MT, RGZ, and KW acquired funding. MR and MT wrote the original draft of the manuscript. MR, AGL, NOV, MFZ, CA, TA, NB, S Greene, STY, MKY, S Guo, EAD, MA, SS, GB, NV, DD, GW, IK, AF, DFE, JN, KW, RGZ, and MT reviewed and edited the manuscript.

## Funding support

This work is the result of NIH funding, in whole or in part, and is subject to the NIH Public Access Policy. Through acceptance of this federal funding, the NIH has been given a right to make the work publicly available in PubMed Central.

- NIH R01DC009645 and R01DC012836 (to MT) and R33AT010408 (to RGZ).
- ANPCyT Argentina PICT-2021 GRF-TI-00422 (to KW).
- American Heart Association postdoctoral fellowship (23POSTCHF1031213 to NV).

## Supplementary Material

Supplemental data

Unedited blot and gel images

Supporting data values

## Figures and Tables

**Table 1:**
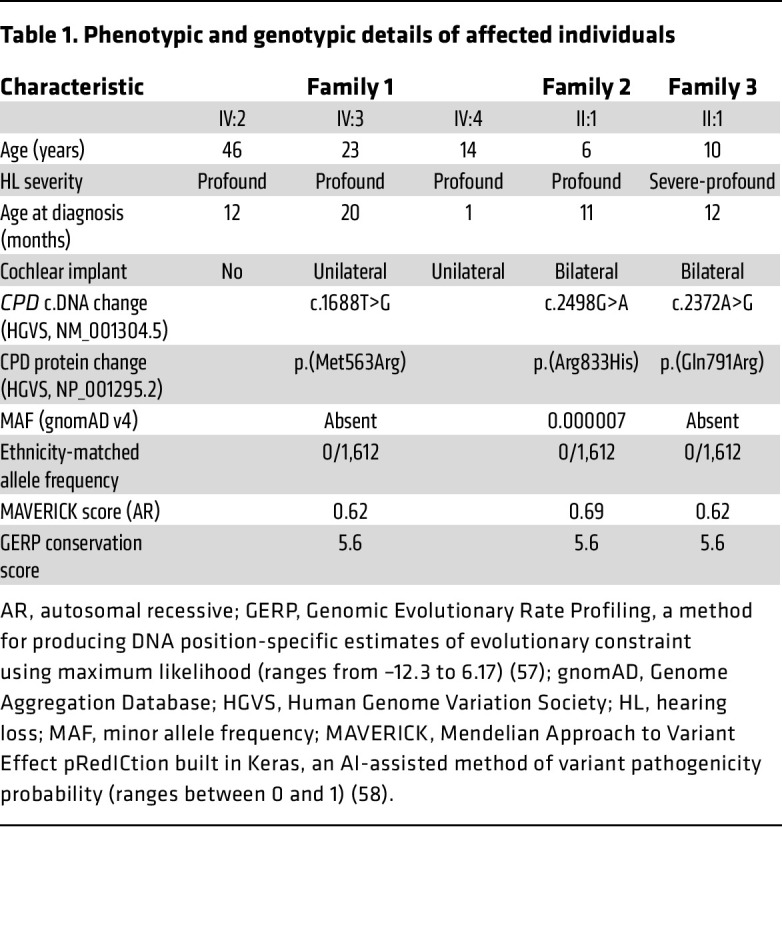
Phenotypic and genotypic details of affected individuals

## Version 1 09/30/2025

In-Press Preview

## Version 2 12/01/2025

Electronic publication
