# Supplementary material for: Carboxypeptidase D deficiency causes hearing loss amenable to treatment
Source: J Clin Invest. 2025 Sep 30;135(23):e192090. doi: 10.1172/JCI192090 (PMC12646673; doi:10.1172/JCI192090)
Supplement: Supplemental data [file jci-135-192090-s065.pdf]

Carboxypeptidase D deficiency causes hearing loss amenable to treatment

Memoona Ramzan[1*], Natalie Ortiz-Vega[2,3*], Mohammad Faraz Zafeer[1*], Amanda G. Lobato[2,3*], Tahir Atik[4], Clemer Abad[1], Nirmal Vadgama[5], Duygu Duman[6,7], Nazım Bozan[8], Enise Avcı Durmuşalioğlu[4], Sunny Greene[2], Shengru Guo[1], Suna Tokgöz-Yılmaz[6], Merve Koç Yekedüz[9,10], Fatma Tuba Eminoğlu[7,9], Mehmet Aydın[8], Serhat Seyhan[11], Ioannis Karakikes[5], Vladimir Camarena[1], Maria Camila Robayo[1], Tijana Canic[3], Güney Bademci[12], Gaofeng Wang[1,12], Amjad Farooq[13], Mei-ling A Joiner[14], Katherina Walz[1,12,15], Daniel F Eberl[14],Jamal Nasir[16], R. Grace Zhai[2,3#], Mustafa Tekin[1,12#]

[1]John P. Hussman Institute for Human Genomics, University of Miami Miller School of Medicine, Miami, FL, USA

[2]Department of Molecular and Cellular Pharmacology, University of Miami Miller School of Medicine, Miami, FL, USA

[3]Department of Neurology, University of Chicago, Chicago, IL, USA

[4]Division of Pediatric Genetics, Department of Pediatrics, School of Medicine, Ege University, Izmir, Türkiye

[5]Department of Cardiothoracic Surgery, Stanford University, Stanford, CA, USA

[6]Department of Audiology, Ankara University Faculty of Health Sciences, Ankara, Türkiye

[7]Ankara University Rare Diseases Application and Research Center, Ankara, Türkiye

[8]Department of Otolaryngology, Faculty of Medicine, Yüzüncü Yıl University, Van, Türkiye

[9]Division of Pediatric Metabolic Diseases, Department of Pediatrics, Ankara University School of Medicine, Ankara, Türkiye

[10]Harvard Medical School, Boston Children's Hospital, Department of Anesthesiology, Critical Care and Pain Medicine, Boston, MA USA

[11]Laboratory of Genetics, Memorial Şişli Hospital, Istanbul, Türkiye

[12]Dr. John T. Macdonald Foundation Department of Human Genetics, University of Miami Miller School of Medicine, Miami, FL, USA

[13]Department of Biochemistry, University of Miami Miller School of Medicine, Miami, FL, USA

[14]University of Iowa, Iowa City, USA

[15]Instituto de Química Biológica de la Facultad de Ciencias Exactas y Naturales (IQUIBICEN) CONICET, Argentina

[16]Division of Life Sciences, University of Northampton, UK

*These authors contributed equally
[#]**Correspondence to:**
Mustafa Tekin
1501 NW 10th Avenue, BRB-610 (M860)
Miami, FL 33136
Phone: +1 (305)243-2381
E-mail: mtekin@med.miami.edu

Or

R. Grace Zhai
5812 South Ellis Avenue
MC 2030, SBRI J310
Chicago, IL 60637
Phone: +1 773-702-6390
E-mail: rgzhai@uchicago.edu

Running Title: *CPD* mutations in deafness

Supplementary Methods

Exome sequencing and bioinformatic analysis

We performed Exome Sequencing (ES) in individuals IV:3 from family 1, II:1 from family 2, and II:1 from family 3; additional genome sequencing (GS) was performed in IV:3 from family 1. The obtained data was aligned with the human reference genome assembly (GRCh37/hg19, UCSC) using the Burrows-Wheeler Aligner (http://bio-bwa.sourceforge.net), and variant calling was performed using FreeBayes (https://github.com/freebayes/freebayes). GENESIS (https://app.tgp-foundation.org) was used for the annotation and variant filtering. The analysis primarily focused on identifying single nucleotide variants, indels, and copy number variants (CNVs) in all known deafness genes retrieved from the hereditary hearing loss homepage (https://hereditaryhearingloss.org/) and OMIM. The OMIM list was filtered by selecting entries with clinical synopses containing keywords deafness, hearing loss, or hard of hearing, and associated with a known molecular cause. Only variants with a minor allele frequency of less than 0.01 from external databases gnomAD (http://gnomad.broadinstitute.org/), and 1000 Genomes Project were selected for further evaluation. We examined homozygous, heterozygous, hemizygous, and compound heterozygous variants. CNV analysis with ES was done with CoNIFER v.02.2 using default parameters. CoNIFER uses a singular value decomposition method to correct systematic biases and identifies a CNV call if the corrected signal reaches a predefined threshold at no less than three consecutive exons (1). We used Manta, Delly and CNVnator for detection of copy number and structural variant detection in GS data (2-4).

The pathogenicity of the variants was assessed according to the criteria provided by the ACMG and ClinGen Hearing Loss Expert Panel (HL-EP), following the ACMG/AMP variant interpretation guidelines (5-7). Additionally, we employed MAVERICK (5), an AI-based tool to

predict the pathogenicity and mode of inheritance of these variants. MAVERICK utilizes artificial intelligence to classify variants based on their effects on the gene product and reported variations within the gene. After the exclusion of variants in all known deafness genes, we focused on those variants with a minor allele frequency less than 0.007 as suggested by the HL-EP Group and CADD>20 in the genes mapped to homozygous regions. Runs of homozygosity were examined in each proband with Enlis Genome Research software (https://www.enlis.com/). Web tools such as GeneMatcher (8) were used to find additional probands with candidate gene/variant.

Genome sequencing alignment to hg38

Genome sequencing raw FASTQ files were uploaded to the Emedgene platform (Emedgene, Illumina®, Inc., CA, USA; www.illumina.com) for secondary and tertiary analysis. The Emedgene platform (v32.0.29), in conjunction with the DRAGEN (v4.0.3) pipeline, was used for read alignment, variant calling, annotation, and runs of homozygosity. Reads were aligned to the GRCh38/hg38 human reference genome. Single nucleotide variants (SNVs), small insertions and deletions (indels), structural variants (SVs), and copy number variants (CNVs) were identified using the DRAGEN pipeline (v4.0.3) (9, 10).

The confirmation and co-segregation of candidate variants in probands and all available family members were performed by Sanger sequencing. For further validation of the association of *CPD* variants with HL, we assessed recessive, rare variants in *CPD* in >1000 ES data of HL families from the internal database (including ethnically matched Turkish controls).

Fibroblast cultures

The dermal fibroblasts were obtained from skin punch biopsies of affected individuals (IV:2 and IV:3 from family 1, II:1 from family 2, and II:1 from family 3), which were brought to the

laboratory under sterile conditions. Fibroblasts were grown with a modification of method described earlier (11). Briefly, each biopsy was cut into 12-15 fine pieces of 4mm and transferred to tissue culture plates with Dulbecco's Modified Eagle Medium (DMEM high glucose, pyruvate, 20% Fetal Bovine Serum, 100 units/mL of Penicillin, 100 μg/mL of Streptomycin and 200 μg/mL of Primocin). The plates were placed in an incubator at 37°C. Media was changed every day until the keratinocytes started migrating out from the biopsies and formed a monolayer. At 50% confluency, we split the cells into T75 cell culture flasks and fed them on alternate days until 90% confluency. Each line was cryopreserved as passage 2 and further studies were conducted using thawed fibroblasts cultured in DMEM with high glucose, 1× pyruvate, 10% Fetal Bovine Serum and 1× antibiotic and antimycotic solution. The control fibroblast cultures HDFa and HDFn were obtained from ATCC (PCS-201-012, PCS-201-010) and were maintained in a similar manner as patient fibroblasts. NHDF-014 and NHDF-018 were commercially grown and obtained from PromoCell, Inc., USA, Lot# 469Z014 and 471Z018.2 respectively. GD016E and GD017E were primary control fibroblasts prepared with explant method as described above.

***Cpd* expression in mice**

Additionally, the expression of Carboxypeptidase D (*Cpd*) in various tissues such as the cochlea, spleen, kidney, and lung were tested at embryos at 18.5 dpc, postnatal day 0 P0, P15, and P30 in wildtype mice. Total RNA was isolated with TRIzol™ Reagent (Thermo Scientific, Cat# 15596026) following the manufacturer's instructions. Before reverse transcription, RNA samples were treated with rDNAse I from DNA-*free*™ kit (Applied Biosystems, USA, Cat# AM1906). cDNA was synthesized using qScript™ XLT cDNA SuperMix (Quanta Biosciences, USA Cat# 95161-100). The primers utilized to amplify a 199bp fragment of the *Cpd* transcripts were as follows: Forward: 5'- TCTCCCGAGCATAAGACCAC -3' and Reverse: 5'-

CTTGCATACCACCTTCCACG – 3’. For *Gapdh,* a 129bp fragment was amplified using the following primers: Forward: 5’-AGGTCGGTGTGAACGGATTTG -3’, and Reverse: 5’-TGTAGACCATGTAGTTGAGGTCA-3’.

***Drosophila* stocks and feeding conditions**

The following fly strains were used in this study: $elav^{C155\text{-}GAL4}$, svr^{1}, *UAS-luciferase RNAi, UAS-silver RNAi* obtained from Bloomington Stock Center. Flies were reared on cornmeal-molasses-yeast medium at 25°C, 50% humidity, with a 12-hour light/dark cycles. For feeding experiments, L-Arginine (Thermo Scientific, Cat# A14730.22) was dissolved in water and then mixed into 10 mL fly food at a final concentration of 400 µg/mL. Sildenafil (Viagra) (Sigma-Aldrich, Cat# SML3033) was dissolved in water and then mixed into 10 mL fly food at a final concentration of 0.886 µg/mL. An equal amount of water was mixed into the fly food as a control. The vials were dried at room temperature for 12 hours before feeding.

Supplementary Figures

Figure S1

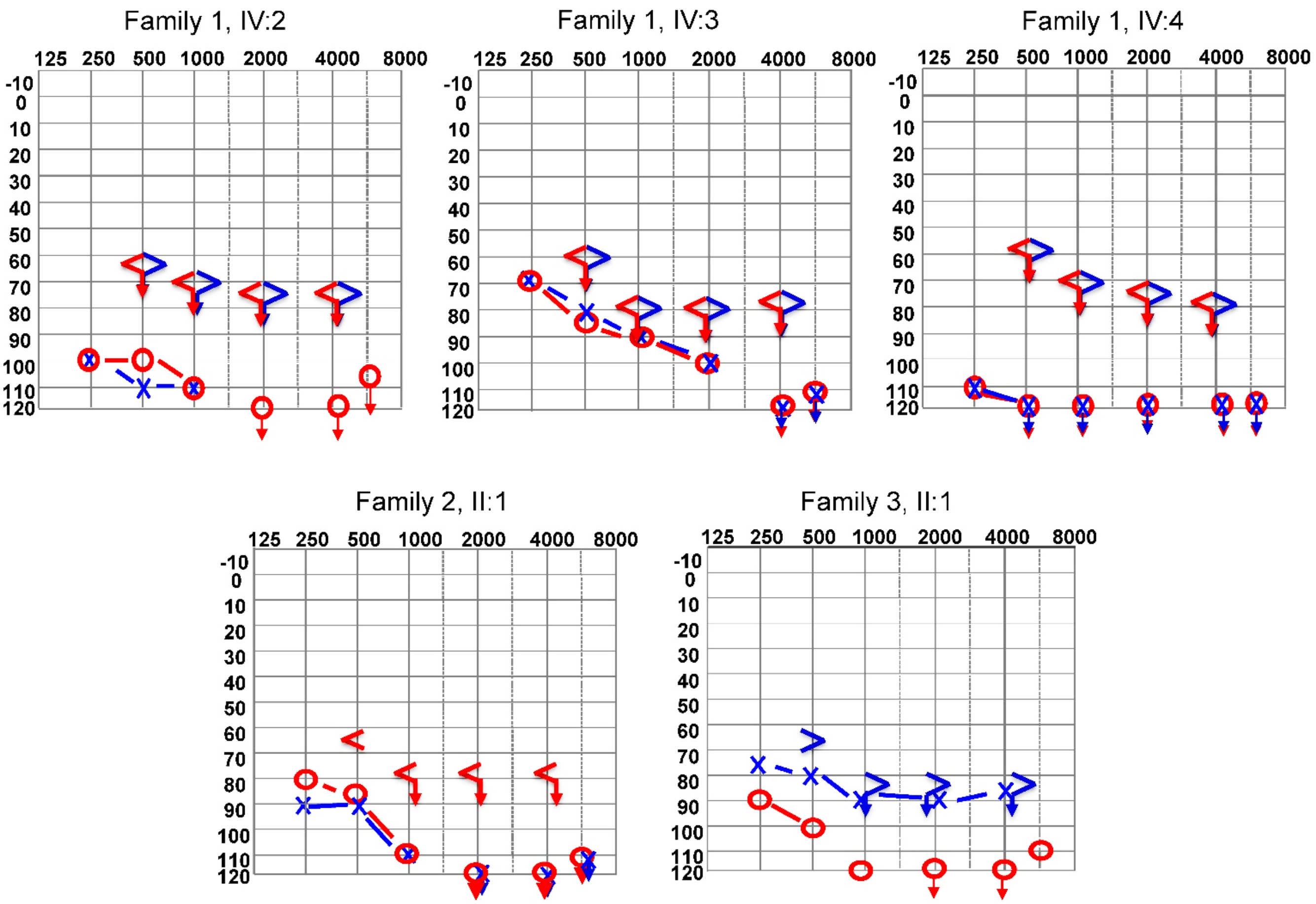

Audiometric evaluation of representative affected individuals from participating families. The audiometric profile of affected individuals in family 1 shows profound hearing loss in three individuals. Individual IV:2 is a female tested at age 46 years, IV:3 is a male and tested at the age of 23 years, and IV:4 is a female aged 14 years. Proband from family 2 is a male evaluated for hearing at the age of 6 years and has profound hearing loss. Proband in family 3 is a male tested at 9 years of age and has severe hearing loss in left and profound hearing loss in the right ear.

-o- air conduction thresholds right ear; **-x-** air conduction threshold left ear; < bone conduction threshold right ear; > bone conduction threshold left ear. Pure tone thresholds were plotted from a response to the given sound in decibel (dB) at different frequencies in Hertz (Hz).

Figure S2

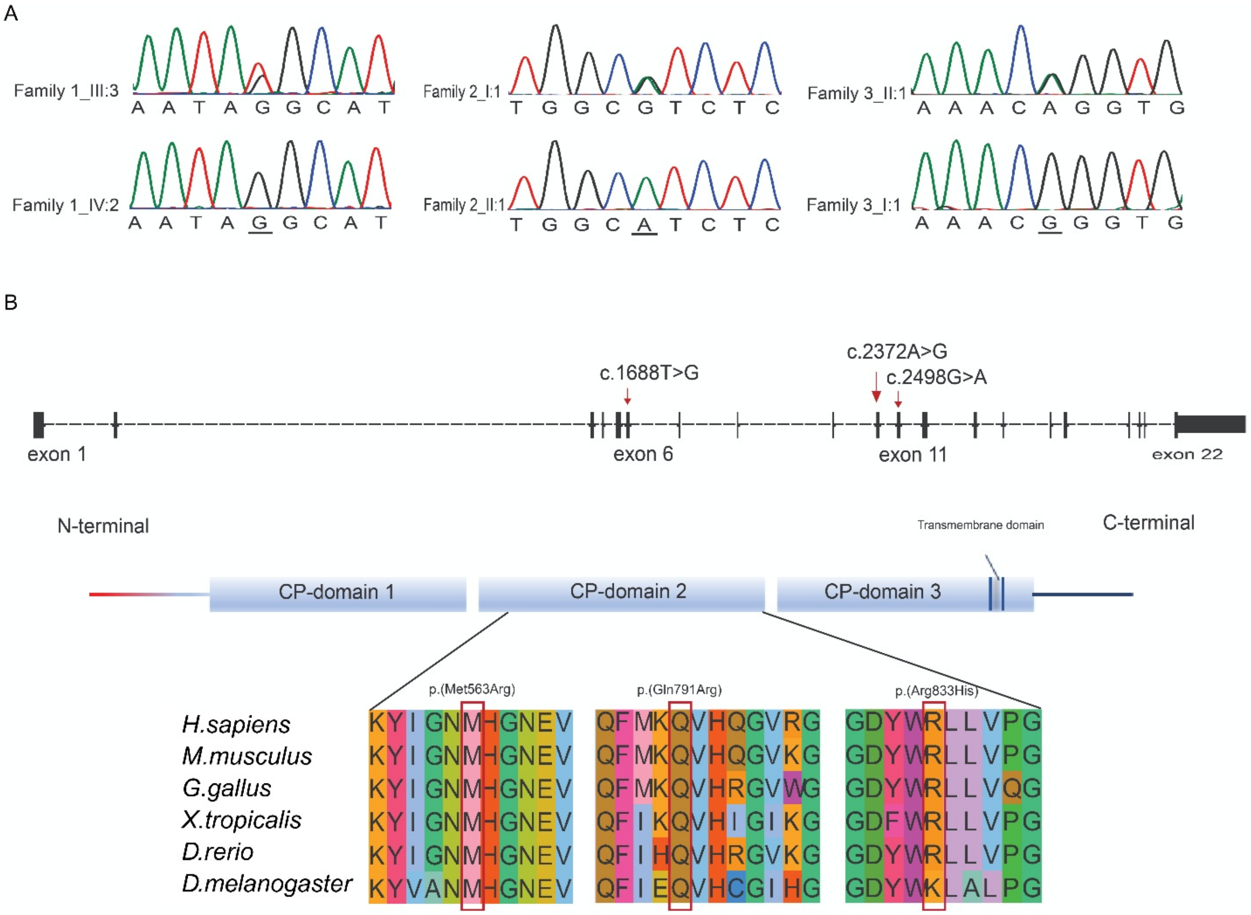

Chromatograms and location of *CPD* variants. (**A)** Partial Sanger traces from each family representing the state of variant c.1688T>G, c.2498G>A and c.2372A>G in respective affected and unaffected individuals. The mutated position is underlined. (**B)** *CPD* is located on chromosome 17 (28705942-28796675 Mbs) (GRCh37) in humans. The identified variants were present in exons 6, 10 and 11 as shown with red arrows. CPD consists of three CP-domains. All the variants in affected individuals are present in a catalytically active CP-domain 2. Partial protein sequence alignment from different species indicates that all the mutations were replacing highly conserved amino acid residues.

Figure S3

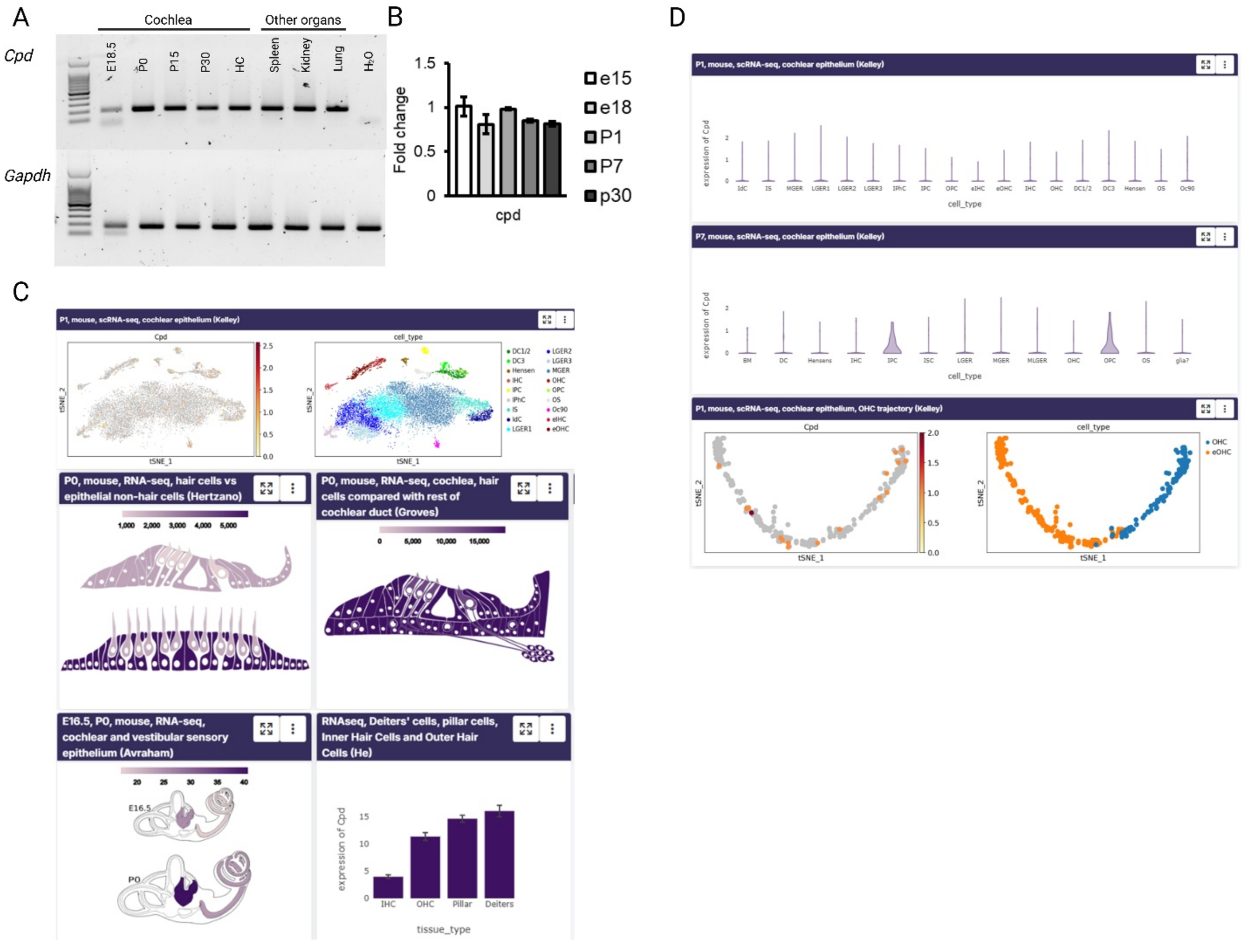

***Cpd* mRNA expression in embryonic and adult mice**. **(A)** RT-PCR analysis of *Cpd* expression in mouse cochleae at various developmental stages (E15.5, E18.5, P0, P15, P30) and in other tissues (heart, liver, spleen, lung, kidney). *Gapdh* serves as a loading control. **(B)** Quantitative real-time PCR analysis showing fold change in *Cpd* mRNA expression in cochlear tissue from embryonic day 15 (E15) to postnatal day 30 (P30), normalized to housekeeping genes. Bars represent mean ± SEM (n = 3 per time point). **(C)** scRNA-seq data from multiple datasets showing *Cpd* expression in cochlear cell types. UMAP plots highlight *Cpd* expression across epithelial and non-epithelial cell populations in the P0 mouse cochlea. Histograms and violin plots display enriched expression in hair cells compared to supporting cells. Additional datasets (e.g., from Avraham and Kolla) confirm *Cpd* expression is highest in Deiters' and pillar cells, followed by inner and outer hair cells. Schematic of the cochlear duct illustrates regions of expression. **(D)** Violin and trajectory plots from the P1 mouse cochlear epithelium scRNA-seq data show dynamic *Cpd* expression across developmental stages and cell clusters. Highest expression is seen in maturing supporting and hair cell populations, with a peak in the Deiters' cell lineage.

HC; Hippocampus, **IHC**: inner hair cells, **OHC**; outer hair cells, **eOHC**; embryonic outer hair cells, **eIHC**; embryonic inner hair cells, **OS**; outer sulcus, **IS**; inner sulcus cells, **ISC**, **IPC**; Inner Pillar Cells, **OPC**; Outer Pillar Cells, **IPhC**;Inner Phalangeal Cells , **DC**; Dieter's cells, **DC ½**;

Dieter Cells from rows 1 and 2, **DC3**; Dieter Cells from row 3, **BM**, **LGER1**, Lateral Greater Epithelial Ridge Cells group 1, **LGER2**, Lateral Greater Epithelial Ridge Cells group 2, **LGER3**, Lateral Greater Epithelial Ridge Cells group 3, **MGER**; Medial Greater Epithelial Ridge Cells, **MLGER**, **IdC**; Interdental Cells, **Oc90**; Cells expressing Oc90.

Figure S4

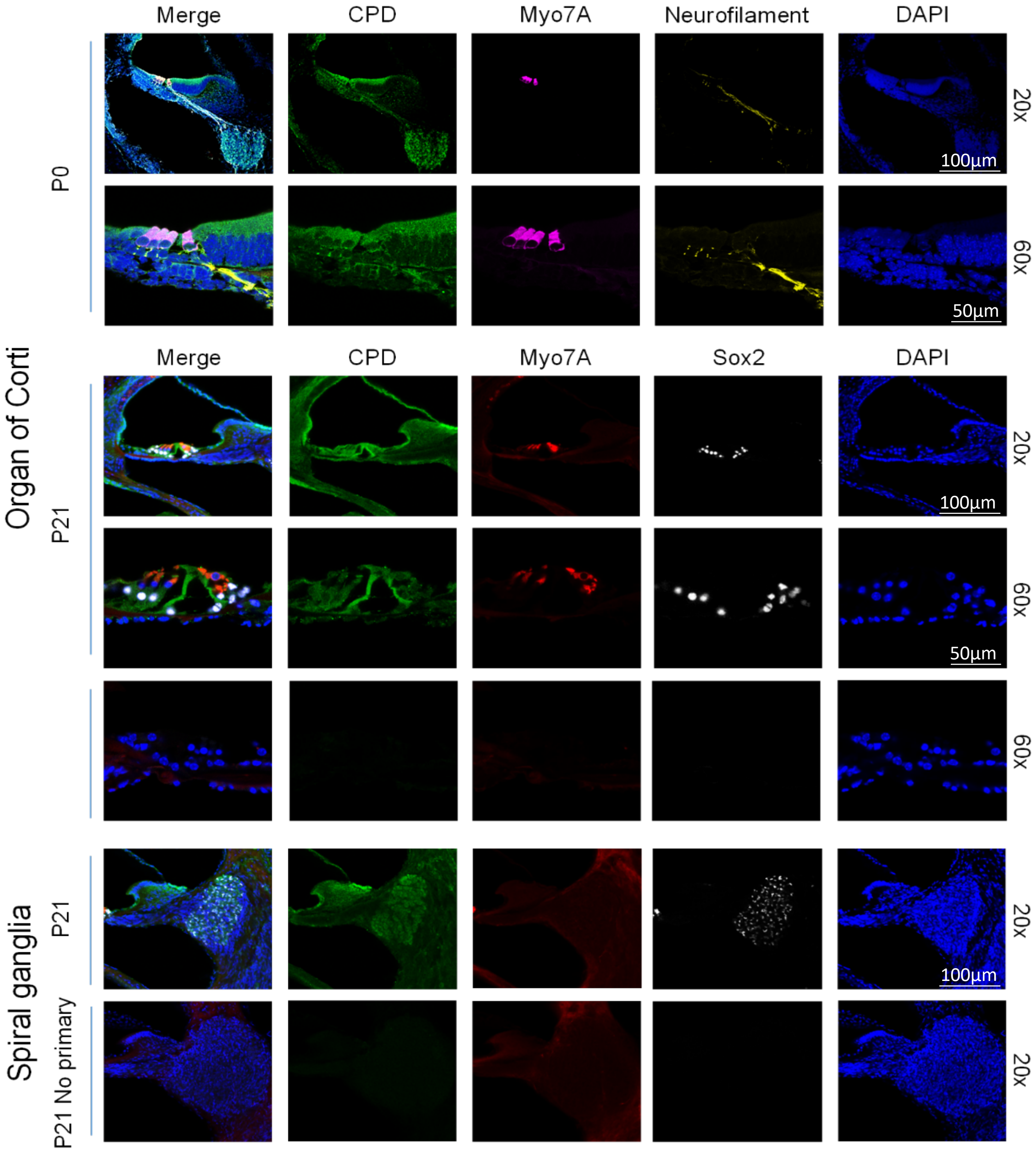
Merge
CPD
Myo7A
Neurofilament
DAPI
P0
20x
100µm
60x
50µm
Organ of Corti
Merge
CPD
Myo7A
Sox2
DAPI
P21
20x
100µm
60x
50µm
60x
Spiral ganglia
P21
20x
100µm
P21 No primary
20x

Localization of CPD in the mouse inner ear at P0 and P21. Immunofluorescence staining showing spatial localization of CPD (green) in the cochlea at postnatal day 0 (P0), postnatal day 21 (P21), and in the spiral ganglia. Hair cells are marked by MYO7A (red), neurons by Neurofilament (yellow), and supporting cells by SOX2 (white). Nuclei are stained with DAPI (blue). Images captured at 20× (scale bar 100µm) and 63× (scale bar 50µm) magnifications. Absence of CPD signal in spiral ganglia at P21 is shown in the "No primary" negative control panel.

Figure S5

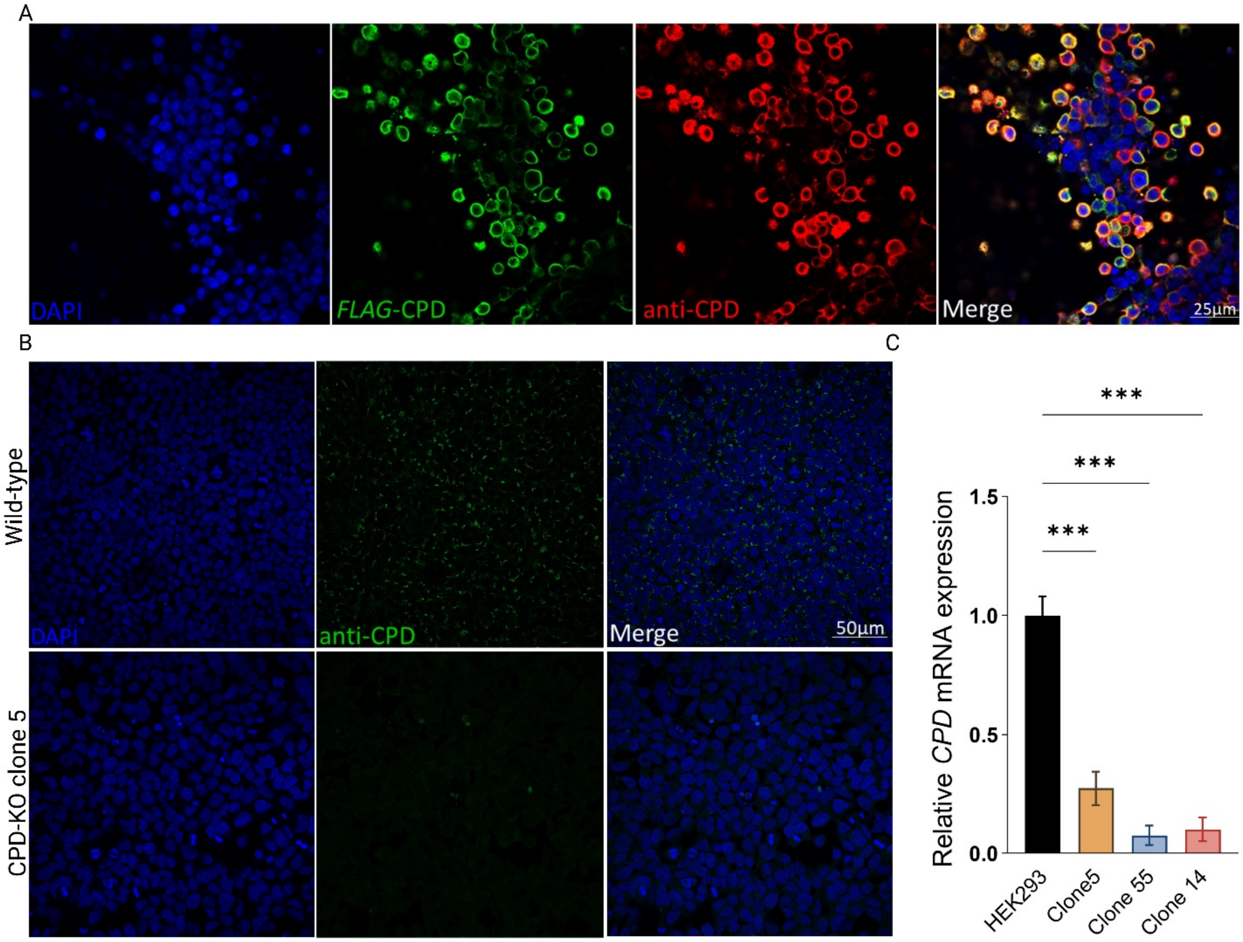

Validation of anti-CPD antibody. (A) Immunofluorescence staining of HEK293 cells transfected with the FLAG-tagged CPD plasmid shows strong colocalization between FLAG-tagged (green) and anti-CPD (red) signals, confirming antibody specificity. Nuclei are counterstained with DAPI. **(B)** Immunostaining of wild-type (top row) and CRISPR-edited CPD knockout (bottom row) HEK293 clones using anti-CPD antibody demonstrates a marked reduction in CPD signal in the representative KO clone, supporting successful gene disruption. **(C)** qRT-PCR analysis of *CPD* mRNA levels in wild-type HEK293 and three independent CRISPR-edited clones (Clone 5, Clone 55, and Clone 14). All knockout clones show significantly reduced *CPD* expression compared to wild-type ($p < 0.001$, unpaired t-test, n = 3 biological replicates).

Figure S6

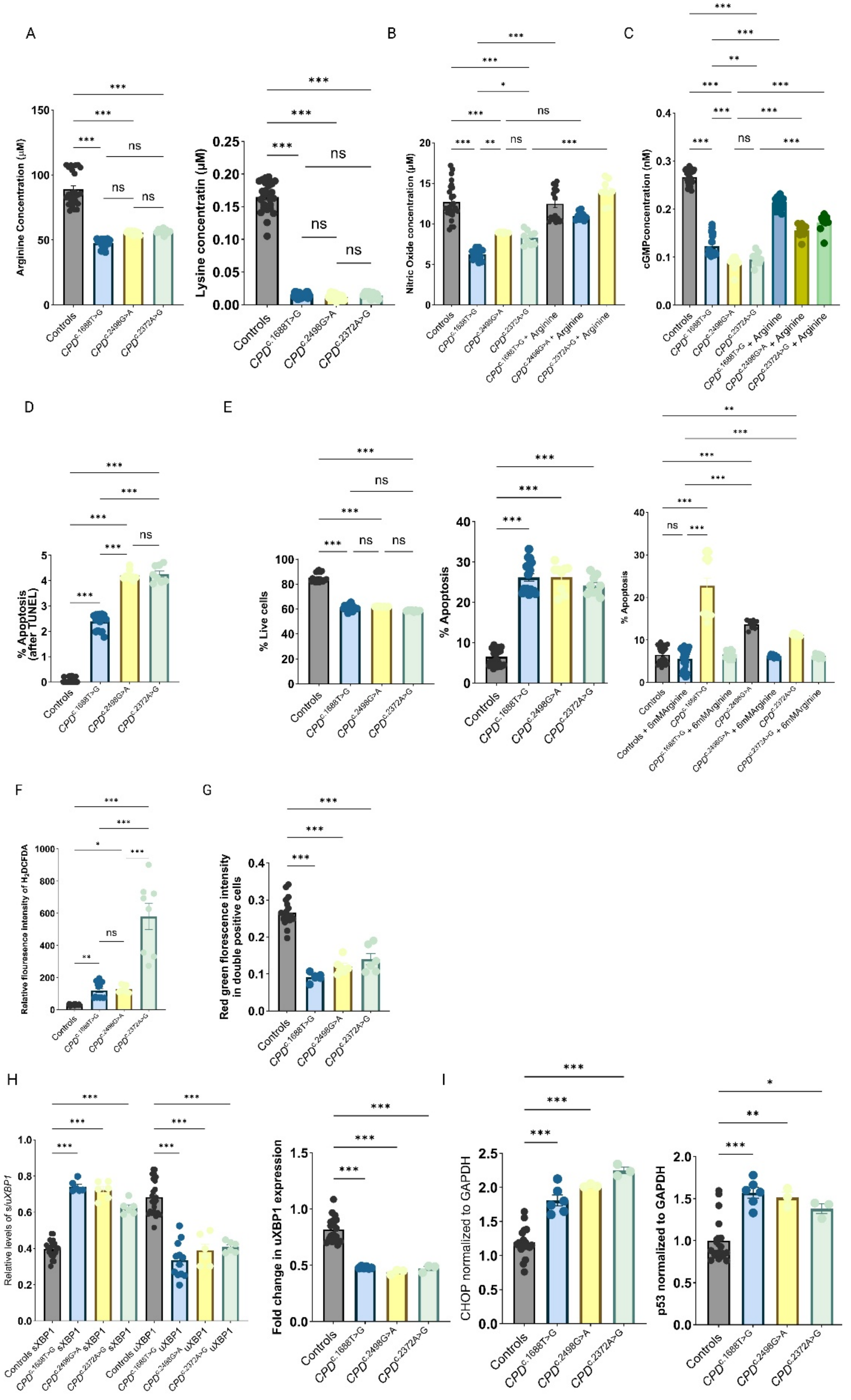
A
B
C
D
E
F
G
H
I
Arginine Concentration (µM)
Lysine concentratin (µM)
Nitric Oxide concentration (µM)
cGMPconcentration (nM)
% Apoptosis (after TUNEL)
% Live cells
% Apoptosis
Relative flouresence Intensity of H2DCFDA
Red green florescence intensity in double positive cells
Relative levels of s/uXBP1
Fold change in uXBP1 expression
CHOP normalized to GAPDH
p53 normalized to GAPDH
Controls
CPD c.1688T>G
CPD c.2498G>A
CPD c.2372A>G
CPD c.1688T>G + Arginine
CPD c.2498G>A + Arginine
CPD c.2372A>G + Arginine
Controls + 6mMArginine
CPD c.1688T>G + 6mMArginine
CPD c.2498G>A + 6mMArginine
CPD c.2372A>G + 6mMArginine
Controls sXBP1
CPD c.1688T>G sXBP1
CPD c.2498G>A sXBP1
CPD c.2372A>G sXBP1
Controls uXBP1
CPD c.1688T>G uXBP1
CPD c.2498G>A uXBP1
CPD c.2372A>G uXBP1
ns

Biochemical and molecular characterization of *CPD* mutant fibroblasts. (A) Quantification of intracellular arginine (left) and lysine (right) concentrations in control and patient-derived fibroblasts with *CPD* variants (c.1688T>G, c.2498G>A, and c.2372A>G). Arginine and lysine levels were significantly reduced in all mutant lines. **(B)** Nitric oxide (NO) concentrations in fibroblasts from controls and *CPD* mutant lines, with or without arginine supplementation. *CPD* mutations led to a marked reduction in NO levels, which were partially rescued by arginine supplementation. **(C)** cGMP levels were significantly decreased in mutant fibroblasts and rescued by arginine treatment. **(D)** Quantification of apoptotic cells using TUNEL staining. Mutant fibroblasts showed significantly increased apoptosis compared to controls. **(E)** % of live cells (left) and apoptotic cells (right) determined by flow cytometry. Apoptosis was elevated in mutant cells and reduced upon arginine supplementation. **(F)** Relative fluorescence intensity of H_2DCFDA as a measure of oxidative stress, which was increased in the fibroblasts with the c.2372A>G variant. **(G)** JC-1 assay showing the red/green fluorescence intensity ratio, indicative of mitochondrial membrane potential. All mutant lines exhibited reduced mitochondrial potential compared to controls. **(H)** Spliced *XBP1* levels (left) and fold change in unspliced *XBP1* expression (right), which are indicative of UPR stress, were significantly elevated in mutant lines compared to controls. **(I)** Western blot quantification of CHOP and p53 protein levels normalized to GAPDH. Both CHOP and p53 were significantly upregulated in *CPD* mutant fibroblasts.Data are presented as mean ± SD. $p < 0.05$ (*), < 0.01 (**), < 0.001 (***), ns = not significant. Statistical comparisons were performed using one-way ANOVA with Tukey's multiple comparisons test.

Figure S7

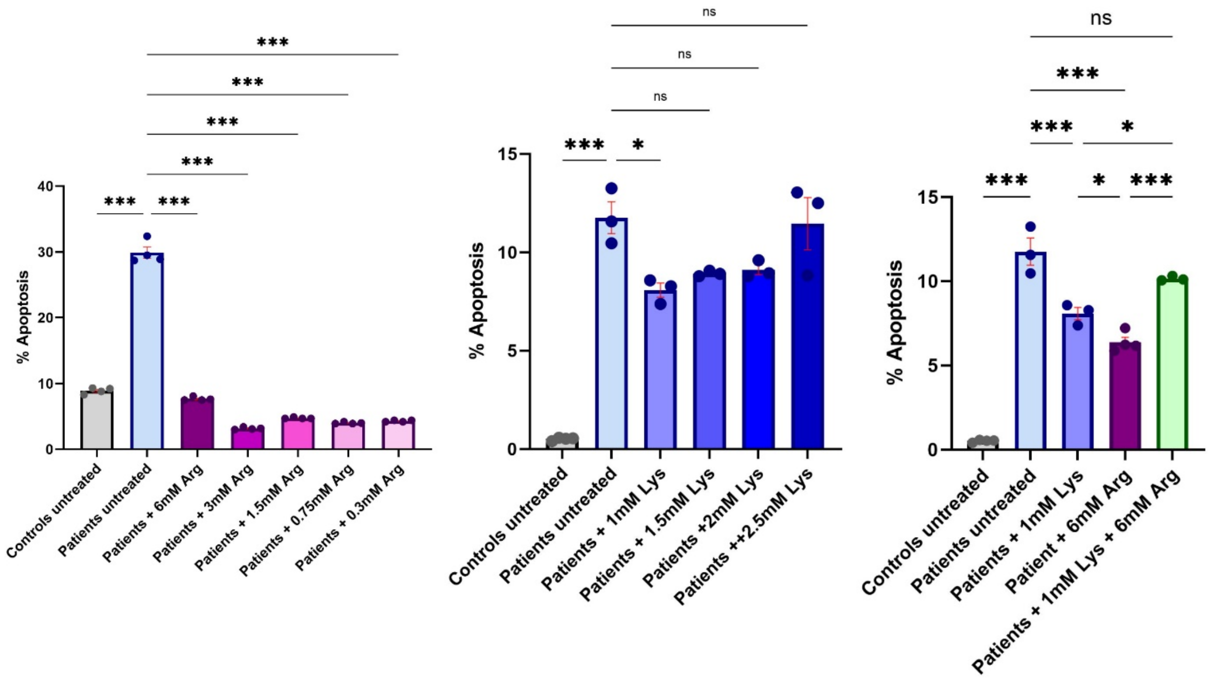

Effect of Arginine and Lysine supplementation on the rate of apoptosis in patient fibroblasts. The left panel shows various concentrations of arginine along with the previously reported concentration for fibroblasts (6mM) (flow cytometry). Arginine has effectively reduced the apoptosis in patient cells. The middle panel (TUNEL assay) shows the effects of different lysine concentrations on apoptosis. The right panel (TUNEL assay) is a comparative analysis of both arginine and lysine supplemented alone and in combination. The doses with the most significant effect on the reduction of apoptosis rate were selected, and the results show that arginine alone is an effective supplement to reduce apoptosis in cells harboring *CPD* mutations. Data are presented as mean ± SEM. Statistical significance was determined by one-way ANOVA. *: $p < 0.05$, **: $p < 0.01$, and ***: $p < 0.001$, ns: not significant

Figure S8

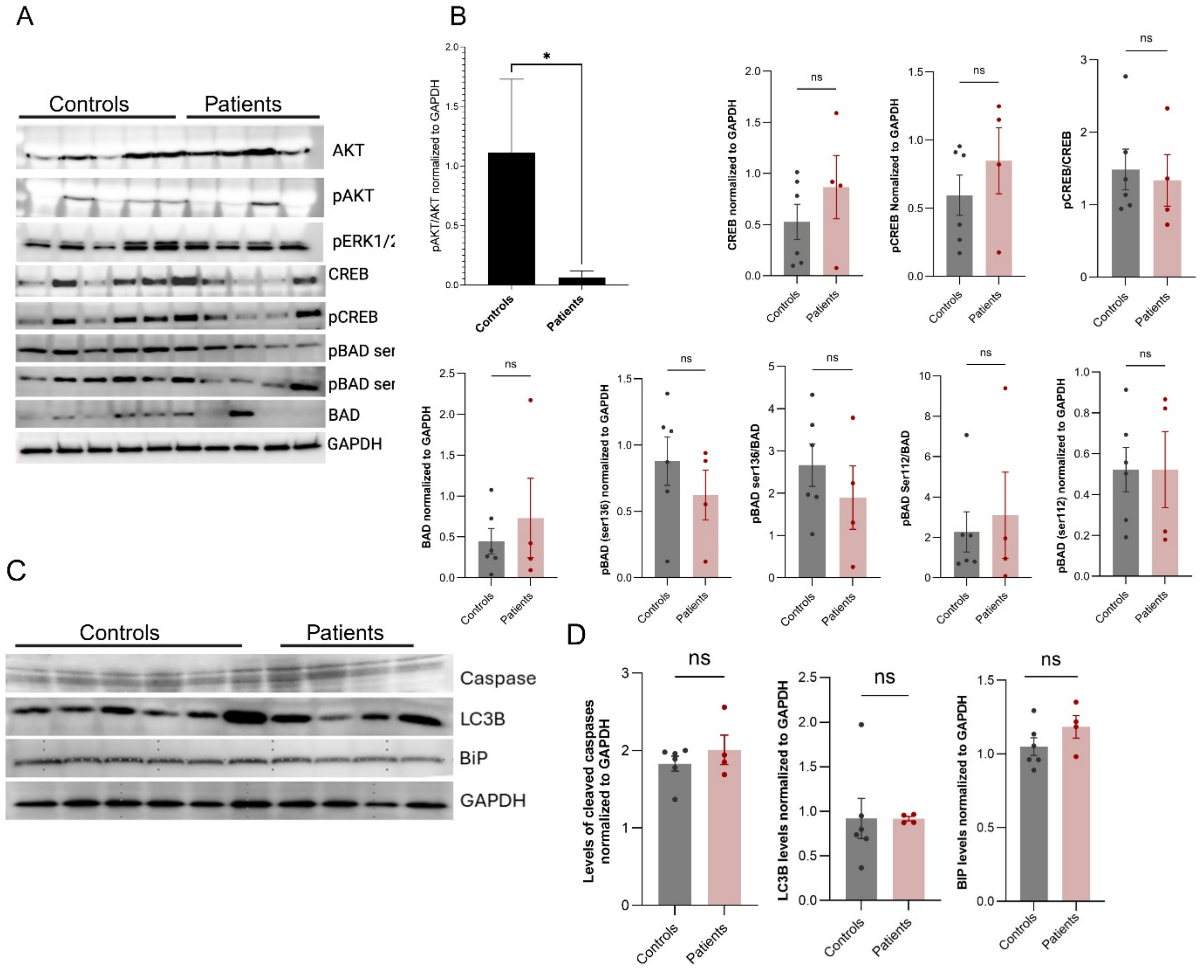

Western blot analysis and quantification of conventional apoptotic and cellular signaling markers in control and patient fibroblasts. (A) Western blot of AKT, phosphorylated AKT (pAKT), pERK1/2, CREB, phosphorylated CREB (pCREB), BAD, phosphorylated BAD (ser136 and ser112), and GAPDH (loading control) to assess conventional pathways potentially involved in apoptosis. **(B)** Quantification of protein levels normalized to GAPDH. Significant reduction in pAKT/AKT ratio was observed in patient samples compared to controls ($*p < 0.05$), while other markers, including total BAD, pBAD, CREB, and pCREB, showed no significant changes (ns). **(C)** Western blot of apoptosis-related proteins, including cleaved Caspase, LC3B (autophagy marker), and BiP (ER stress marker), with GAPDH as the loading control. **(D)** Quantification of Caspase, BiP and LC3B levels normalized to GAPDH. The levels of all these conventional markers showed no significant differences among patient and control samples.

Figure S9

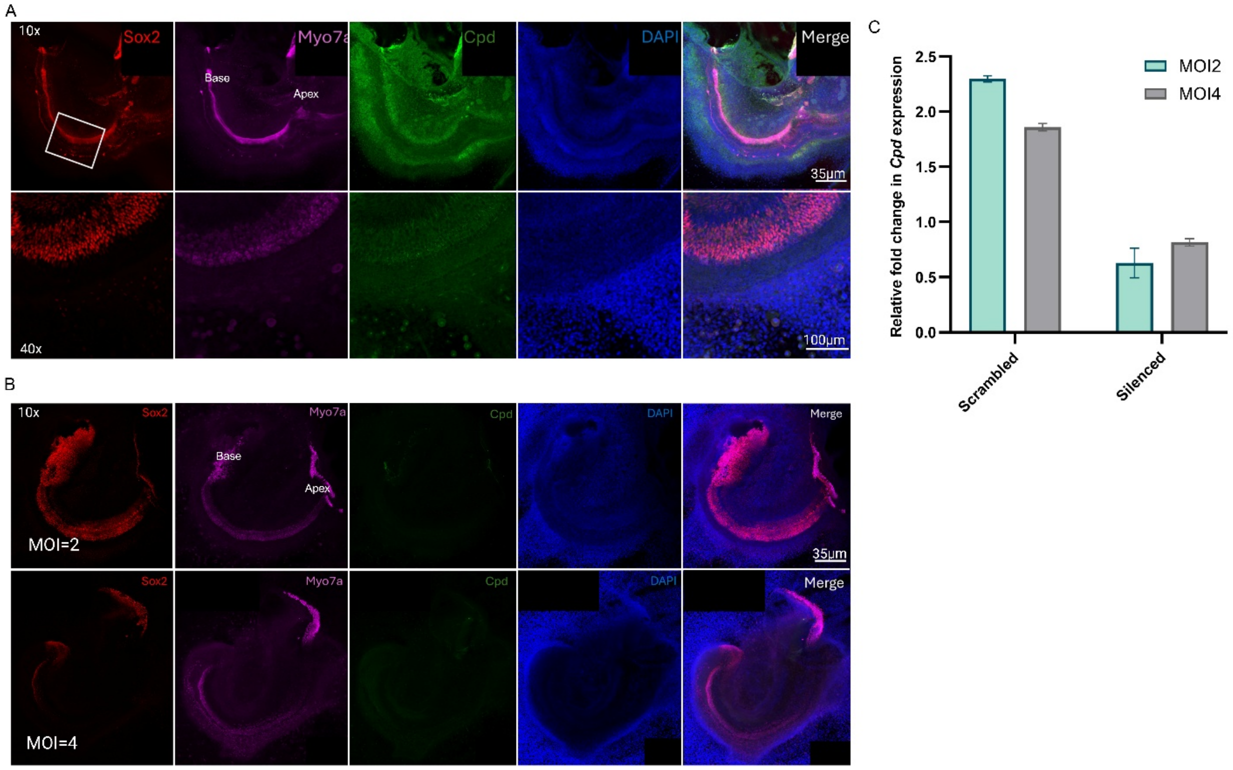

Cpd siRNA lentivirus significantly reduces gene expression. (A) Representative confocal images (10x; scale bar 35µm) of explants transduced with scrambled siRNA (control) at a multiplicity of infection (MOI) of 2×10^6. The lower panel is the closer image (40x; scale bar 100µm) of the area indicated with the white rectangle. **(B)** The cochlear explant infected with an MOI of 2×10^6 is the most effective dose, as seen by the low signal of CPD (green). **(C)** qRT-PCR also shows a decrease in expression of *Cpd* relative to the scrambled siRNA. Data are represented as mean ± SEM (n = 4 cochleae per condition from three different litters). Statistical significance was determined using a two-way ANOVA, followed by multiple comparisons. ***$p \leq 0.001$.

Figure S10

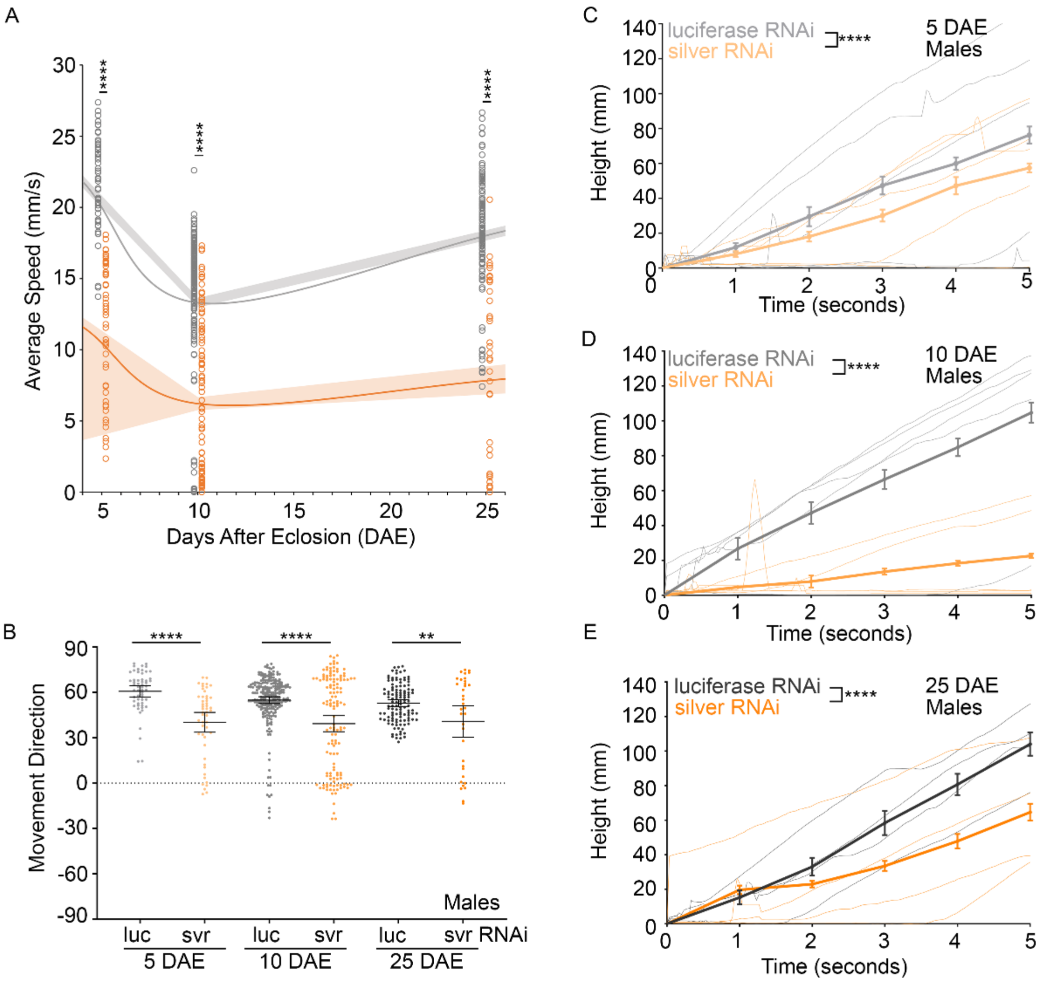

***silver* loss of function results in impaired negative geotaxis behavior. (A-E)** Negative geotaxis analysis of pan-neuronal knockdown of *luciferase* (gray) or *silver* (orange) in male flies. Assessment of average speed **(A)**, movement direction **(B)**, climbing distance at 5 DAE for luciferase (n = 56) or silver (n = 50) **(C)**, climbing distance at 10 DAE for *luciferase* (n = 250) or *silver* (n = 166) **(D)**, and climbing distance at 25 DAE for luciferase (n = 120) or silver (n = 39) **(E)**.

Figure S11

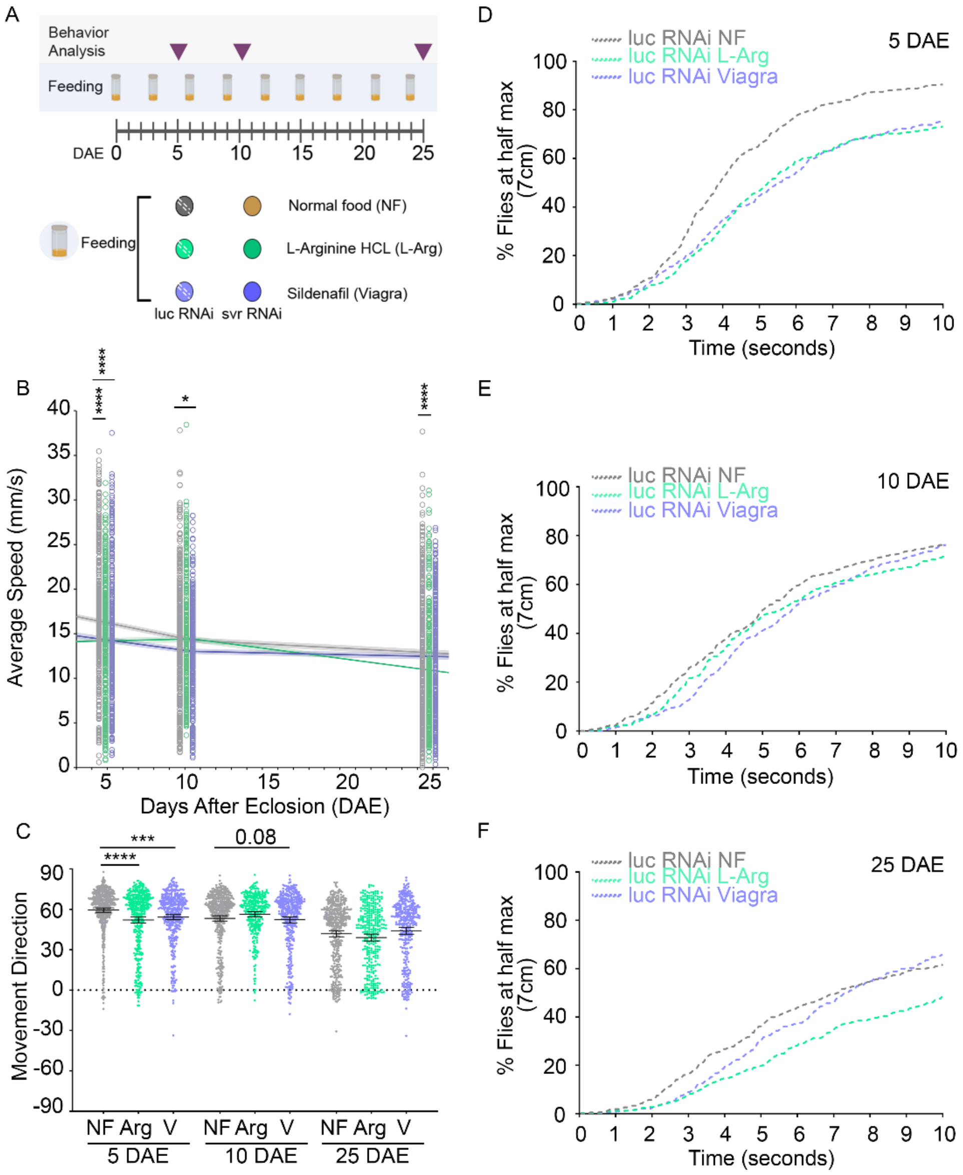

Feeding with L-Arginine or sildenafil showed apparent mild toxicity in *luciferase* RNAi control flies. (A) Feeding paradigm for Normal food (NF), L-Arginine (L-Arg), and sildenafil (Viagra). **(B-F)** Negative geotaxis behavior for pan-neuronal knockdown of *luciferase* females (*elav*$^{C155\text{-}GAL4}$*>UAS- luciferase RNAi*) fed with either NF (gray) (n = 470), L-Arg (green) (n = 400) or Viagra (blue) (n = 390). Assessment of average speed **(B)**, movement direction **(C)**, and percentage of flies to reach 7cm in 10 seconds for 5 DAE **(D)**, 10 DAE **(E)** and 25 DAE **(F)**. Data are presented as mean + 95% CI. *p<0.05, **p<0.01, ***p<0.001, ****p<0.0001.

Figure S12

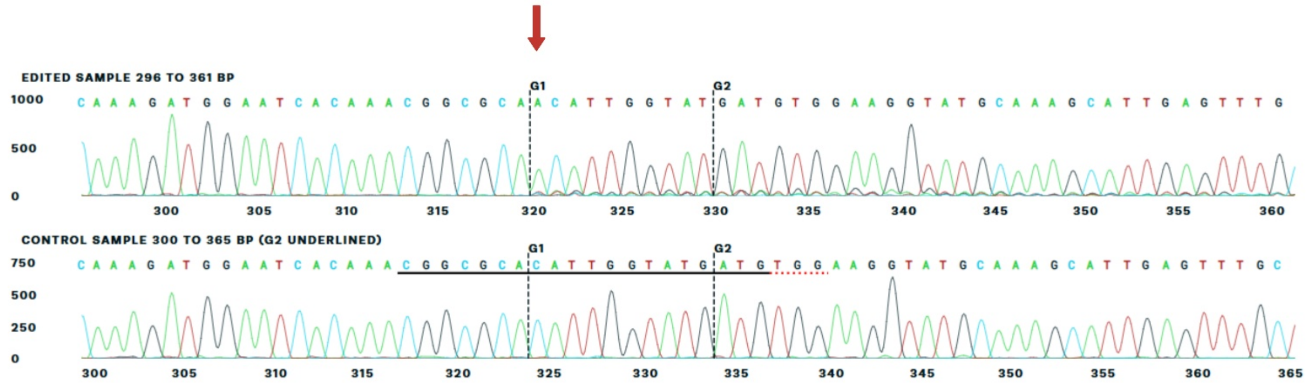

Representative Sanger sequencing chromatograms of the *CPD* gene from CRISPR/Cas9 edited HEK293 cells (top) and a wild-type control (bottom). The red arrow indicates an insertion of a single adenine (A) nucleotide, resulting in a frameshift mutation in the edited sample. G1 and G2 represent the cut site.

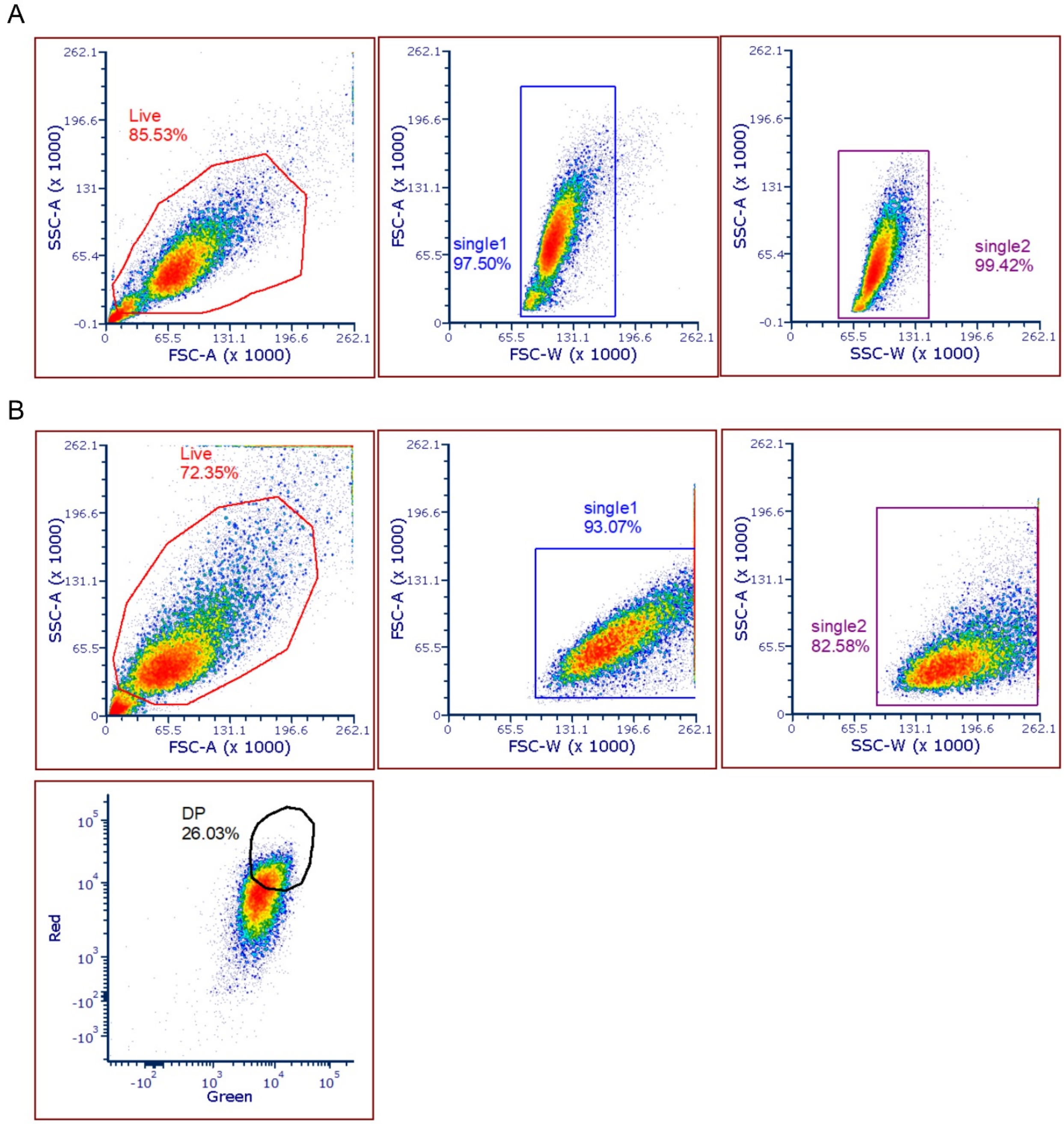

Figure S 13: Flow cytometry gatings. **(A)** Annexin V and PI staining assay shown in Figure 3b. **(B)** JC-1 staining assay shown in Figure 3e.

Supplementary Tables

Table S1

Additional details on families with *CPD* variants.

Characteristic	Family 1			Family 2	Family 3
	IV:2	IV:3 (1593-102)	IV:4 (1593-101)	II:1 (2764-101)	II:1 (2161-101)
**Sequencing Method used**	Sanger sequencing	ES / GS	GS	ES / GS	ES / GS
**Sequencing Capture method (ES)**	-	Agilent SureSelect Human All Exon kit V5	-	IDT xGen V2	IDT xGen V2
**Sequencing Instrument Used**	ABI XL	Illumina HiSeq 2000 / NovaSeq 6000	NovaSeq 6000	BGI-SEQ500/NovaSeq X	BGI-SEQ500/ NovaSeq X
**ES Average read depth**	-	51.98x	-	131.10x	131.35
**GS Average read depth**		28.8x	43.1x	32.7x	35.3x
**GS Coverage for >10x read depth**		93.9%	94%	94%	94.3%
***CPD* variant genomic coordinates (HGVS, NC_000017.11) (hg38)**		chr17:30423536	chr17:30423536	chr17:30443926	chr17:30442449
**Homozygous runs flanking *CPD* variants (hg38)**	-	chr17:14338913-33099137	chr17:14338913-33099137	chr17:13910213-34012642	chr17:15158576-56911778
**Height SDS**	-1	-2.2	-1.4	-1.5	-0.3
**BMI Z score**	1.08	-0.99	-1.27	-1.96	0.06
**Communication**	Sign language	Oral	Oral	Oral	Oral
**Temporal bone CT/MRI**	N/A	Normal	Normal	Normal	Normal
**Parental hearing (F/M)**	Normal/Normal	Normal/Normal		Moderate HL/Normal	Normal/Normal
**Plasma arginine (31-132 µmol/L)**	N/A	38.67	59.80	60.65	N/A
**Plasma lysine (59-240 µmol/L)**	N/A	191.58	215	185.03	N/A
**Plasma ornithine (38-130 µmol/L)**	N/A	94.84	80.49	60.41	N/A
**Plasma citrulline (17-46 µmol/L)**	N/A	25.17	23.24	32.40	N/A
**Zinc (serum/plasma) µg/dL(60-120)**	92	89	89	80	82

ES: Exome sequencing **GS:** Genome Sequencing, **N/A**: Not Available, **F:** Father, **M:** Mother, **HL:** Hearing Loss

Table S2

Systemic clinical features of individuals with *CPD* variants

Clinical Feature / Measurement		Family 1			Family 2	Family 3
		**IV:2**	**IV:3**	**IV:4**	**II:1**	**II:1**
**Age/sex**		46/F	23/M	14/F	6/M	10/M
**Cardiovascular**	History of Hypertension	No	No	No	No	No
	Blood pressure (mmHg)	N/A	115/75	100/50	70/40	105/70
	Heart rate (bpm)	N/A	88	77	90	75
	Pulmonary hypertension (echocardiography)	N/A	N/A	N/A	No	No
	History of ischemic events (MI or stroke)	No	No	No	No	No
**Neurological**	Global developmental delay	No	No	No	No	No
	Migraine	No	No	No	N/A	No
	Neurodegenerative disorder	No	No	No	No	No
**Immune system**	Recurrent infections	No	No	No	No	No
	Chronic inflammation	No	No	No	No	No
**Renal**	Serum sodium	Normal	Normal	Normal	Normal	Normal
	Kidney function tests (BUN/Cre/GFR)	Normal	Normal	Normal	Normal	Normal
**Gastrointestinal**	GI motility abnormalities	N/A	N/A	N/A	N/A	None
**Hematologic**	History of thrombosis	No	No	No	No	No
	Platelet count	N/A	N/A	N/A	N/A	Normal
	Bleeding tendency / coagulopathy	No	No	No	No	No

Table S3

SDM primers

Primer Name	Primer Sequence (5' to 3')
t1688g_F	5'-cacttcatttccatgcctatttccaatgtacttaaattctggttcacc-3'
t1688g_R	5'-ggtgaaccagaatttaagtacattggaaataggcatggaaatgaagtg-3'
a2372g_F	5'-gacgccctgatgaacccgtttcataaactggattagt-3'
a2372g_R	5'-actaatccagtttatgaaacgggttcatcagggcgtc-3'
g2498a_F	5'-ttcctggaaccaagagatgccagtaatctccagtt-3'
g2498a_R	5'-aactggagattactggcatctcttggttccaggaa-3'

References

1. Krumm N, Sudmant PH, Ko A, et al. Copy number variation detection and genotyping from exome sequence data. Genome Res. 2012;22(8):1525-32.

2. Chen X, Schulz-Trieglaff O, Shaw R, et al. Manta: Rapid detection of structural variants and indels for germline and cancer sequencing applications. Bioinformatics. 2016;32(8):1220-2.

3. Rausch T, Zichner T, Schlattl A, et al. DELLY: structural variant discovery by integrated paired-end and split-read analysis. Bioinformatics. 2012;28(18):i333-i9.

4. Abyzov A, Urban AE, Snyder M, et al. CNVnator: an approach to discover, genotype, and characterize typical and atypical CNVs from family and population genome sequencing. Genome Res. 2011;21(6):974-84.

5. Richards S, Aziz N, Bale S, et al. Standards and guidelines for the interpretation of sequence variants: a joint consensus recommendation of the American College of Medical Genetics and Genomics and the Association for Molecular Pathology. Genet Med. 2015;17(5):405-23.

6. Oza AM, DiStefano MT, Hemphill SE, et al. Expert specification of the ACMG/AMP variant interpretation guidelines for genetic hearing loss. Hum Mutat. 2018;39(11):1593-613.

7. Tavtigian SV, Harrison SM, Boucher KM, et al. Fitting a naturally scaled point system to the ACMG/AMP variant classification guidelines. Hum Mutat. 2020;41(10):1734-7.

8. Sobreira N, Schiettecatte F, Valle D, et al. GeneMatcher: A matching tool for connecting investigators with an interest in the same gene. Hum Mutat. 2015;36(10):928-30.

9. Meng L, Attali R, Talmy T, et al. Evaluation of an automated genome interpretation model for rare disease routinely used in a clinical genetic laboratory. Genet Med. 2023;25(6):100830.

10. Behera S, Catreux S, Rossi M, et al. Comprehensive and accurate genome analysis at scale using DRAGEN accelerated algorithms. bioRxiv [Preprint]. 2024.

11. Vangipuram M, Ting D, Kim S, et al. Skin punch biopsy explant culture for derivation of primary human fibroblasts. J Vis Exp. 2013(77):e3779.
