# Supplementary material for: Carboxypeptidase D deficiency causes hearing loss amenable to treatment
Source: J Clin Invest. 2025 Sep 30;135(23):e192090. doi: 10.1172/JCI192090 (PMC12646673; doi:10.1172/JCI192090)
Supplement: Unedited blot and gel images [file jci-135-192090-s066.pdf]

Spliced and un-spliced forms of *XBP1* amplified from patients and control fibroblasts

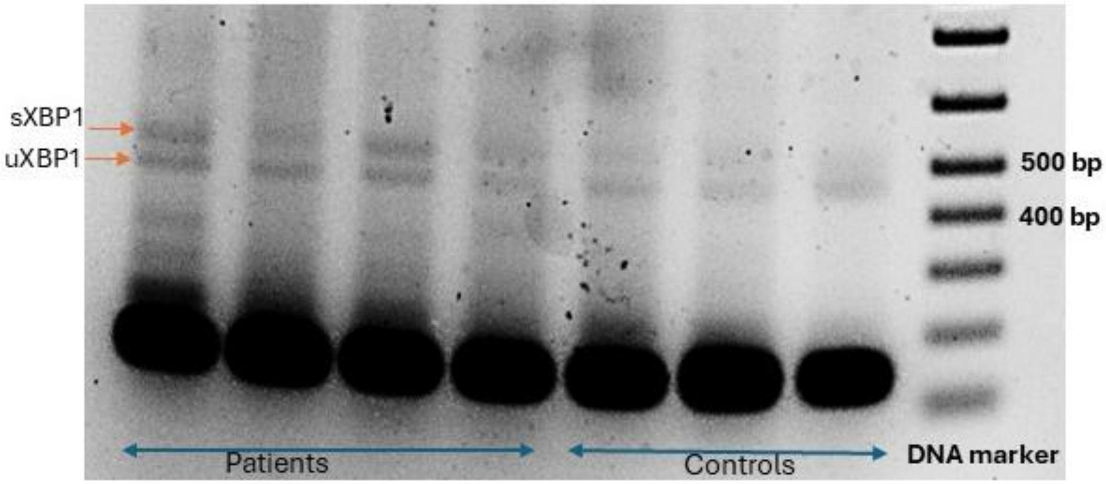

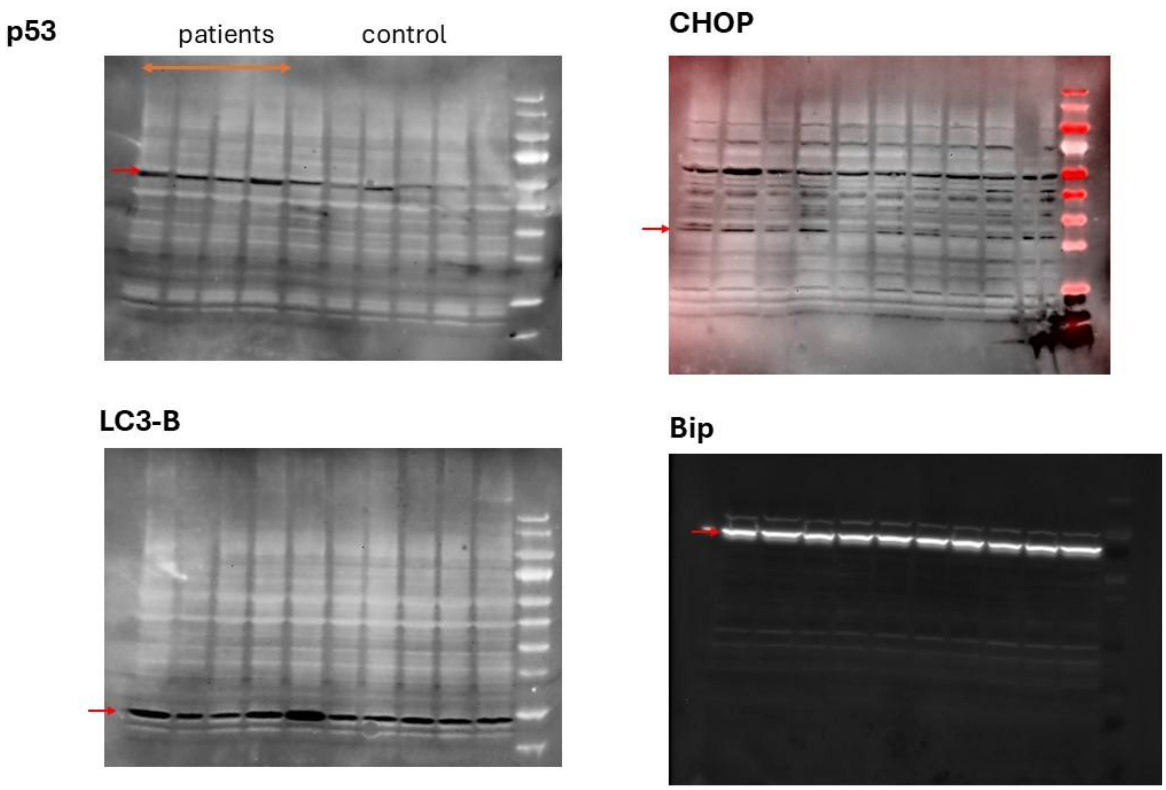
p53
patients
control
CHOP
LC3-B
Bip
